# Innovative Plant-Based Nutraceuticals: Enhancing Iron Bioavailability to Address Iron Deficiency Anaemia

**DOI:** 10.3390/antiox14111335

**Published:** 2025-11-05

**Authors:** Nemanja Živanović, Vesna Mijatović Jovin, Bojana Andrejić Višnjić, Diandra Pintać Šarac, Danica Ćujić, Nataša Simin, Marija Lesjak

**Affiliations:** 1 Department of Chemistry, Biochemistry and Environmental Protection, Faculty of Sciences, University of Novi Sad, Trg Dositeja Obradovica 3, 21000 Novi Sad, Serbia; nemanja.zivanovic@dh.uns.ac.rs (N.Ž.); natasa.simin@dh.uns.ac.rs (N.S.); 2 Faculty of Medicine, University of Novi Sad, Hajduk Veljkova 3, 21137 Novi Sad, Serbia; vesna.mijatovic-jovin@mf.uns.ac.rs (V.M.J.); bojana.andrejic-visnjic@mf.uns.ac.rs (B.A.V.); diandra.pintac@mf.uns.ac.rs (D.P.Š.); 3 Institute for the Application of Nuclear Energy, University of Belgrade, Banatska 31b, 11080 Zemun, Serbia; danicac@inep.co.rs

**Keywords:** iron deficiency anaemia, bioavailable iron, plant-based nutraceuticals, iron absorption inhibitors, animal studies

## Abstract

Iron deficiency anaemia (IDA) affects 25% of the global population, with detrimental effects on the health of women and children. Treatments with iron supplements offer temporary relief but often yield adverse effects, hindering patient adherence. Additionally, IDA is associated with oxidative stress, which becomes significantly exacerbated during iron supplementation. Our study aimed to address this challenge by developing a plant-based nutritional formula rich in bioavailable iron and antioxidants devoid of adverse effects. Chemical analysis of edible plants, focused on the content of iron and iron absorption inhibitors, guided formula development. In vivo studies on rats with IDA evaluated iron bioavailability from the formulated plant-based nutraceuticals. Results of animal studies showed significant improvements in IDA-associated blood parameters after 28 days of oral administration of the nutraceuticals. Additionally, the nutraceuticals did not impede the benefits of iron supplementation. These findings strongly indicate that plant-based nutraceuticals can serve as an effective source of bioavailable iron, potentially improving treatment adherence and at the same time aligning with ongoing WHO and UNICEF initiatives to enhance IDA management.

## 1. Introduction

Iron deficiency anaemia (IDA) affects almost a quarter of the world’s population. IDA predominantly affects women of reproductive age and children, with approximately 30% of non-pregnant women aged 14–49 facing IDA globally. Alarmingly, 37% of pregnant women and 40% of children aged 6–59 months worldwide suffer from IDA [1,2].

The consequences of IDA are severe, especially during pregnancy, contributing to restricted foetal growth, low birth weight, developmental impairments, and increased risks of premature birth. IDA in infancy leads to long-term cognitive, motor, and social–emotional deficits [3,4,5,6,7,8]. In adults, IDA diminishes cognitive and physical capacities, reduces work productivity, and adversely affects economies [9,10,11,12]. Bearing in mind the scale of the problem, the World Health Organisation (WHO) has set a WHO’s Global Nutrition Target to reduce IDA in women of reproductive age by 50% by 2030 [13,14].

IDA stems from insufficient iron levels in the blood or tissue, often due to blood loss, increased iron demands during pregnancy and childhood growth spurts, or inadequate iron intake or absorption from diet [1,15,16]. Interestingly, there is growing evidence that IDA may be associated with, and potentially intensified by, elevated levels of oxidative stress. Under hypoxic conditions characteristic of IDA, the production of reactive oxygen species (ROS) is increased, while the efficiency of antioxidant defence systems is diminished, leading to oxidative stress. This is supported by findings of elevated biomarkers of oxidative damage, such as malondialdehyde (MDA), nitric oxide (^•^NO), total peroxide levels, and an increased oxidative stress index. Concurrently, levels of key antioxidants, including total antioxidant capacity, catalase, glutathione peroxidase (GPx), glutathione reductase (GR), and vitamins C and E, are significantly reduced [17,18,19].

Among all the mentioned causes of IDA, only the iron absorption rate can be managed [13]. IDA is typically treated with orally administered artificial iron supplements, but they often require high doses and long durations, leading to unpleasant side effects like upset stomach and constipation. These side effects, coupled with the fasting conditions necessary for administration, result in poor compliance, especially among high-risk groups like children and pregnant women [20,21,22]. Furthermore, evidence indicates that iron supplementation may induce oxidative damage. This effect appears to be minimal with oral administration but is more pronounced following intramuscular or intravenous administration, where iron concentrations are significantly higher [23].

Alternatively, improving iron absorption through diet, especially with bioavailable sources like red meat, poultry, fish, and eggs, is another approach. However, traditional advice to consume iron-rich plant foods (e.g., spinach, broccoli, raisins, apricots, berries, etc.) is often ineffective due to their high content of iron absorption inhibitors like polyphenols and phytic acid. Despite the richness of iron in these foods, iron bioavailability is limited, hindering IDA treatment efficacy [13,24,25,26].

The negative impact of certain dietary polyphenols, particularly those with hydroxyl group positions that enable effective iron chelation, on iron bioavailability in vivo has been documented in a couple of dozen studies over the past four decades [27,28]. It is shown that polyphenol-rich foods like tea, wine, coffee, and different vegetables greatly inhibit iron absorption. Phytic acid, also widespread in plants, is known to significantly hinder iron absorption [29,30,31]. For example, the addition of 50 mg of bean polyphenols to a meal reduces iron absorption by 14%, and the addition of 200 mg inhibits it by 45% [32]. Moreover, polyphenols such as tannins, which are present in tea and coffee, also have an inhibitory effect on iron absorption from food [33]. Phytic acid has an even stronger inhibitory effect on iron absorption, since even 2–10 mg per meal can result in significantly lower iron bioavailability [34]. These inhibitory effects of phytic acids and polyphenols can be overcome by the iron absorption-promoting effects of vitamin C. For example, for a meal containing over 100 mg of tannic acid, 50 mg of vitamin C would be needed to overcome the inhibitory effect; and for a meal containing up to 60 mg of phytic acid, 30 mg of vitamin C would be needed to overcome the inhibitory effect. Vitamin C acts as a potent reducing agent, enhancing iron bioavailability by converting ferric iron (Fe^3+^) to the more readily absorbed ferrous form (Fe^2+^), thereby improving intestinal iron uptake [35].

On the other hand, antioxidant components commonly found in fruits and vegetables, such as vitamin C and certain polyphenols, not only play a role in modulating iron bioavailability but also help reduce oxidative stress associated with IDA. Both vitamin C and polyphenols exhibit potent antioxidant properties, capable of scavenging ROS generated during IDA and iron supplementation. These effects contribute to a reduction in iron-induced oxidative damage, thereby protecting cellular structures and potentially improving clinical outcomes in individuals with IDA [Deng]. Importantly, polyphenols that contain hydroxyl groups positioned such that they do not effectively chelate iron may offer dual benefits in the context of IDA. They are unlikely to impair iron absorption and also have antioxidative effects, which are particularly valuable given the elevated oxidative stress observed in IDA.

To address all problems listed above, the WHO listed recommended interventions for the prevention and treatment of IDA, stating that the design of new diets containing adequate amounts of bioavailable iron should be a global goal [2,13].

In order to follow these recommendations, this study aimed to develop a nutritional formula made of various plant-based foodstuffs, according to which different nutraceuticals rich in highly bioavailable iron, such as juices, smoothies, purees, bars, soups, ice creams, etc., could be made.

Detailed chemical analysis of edible plants, focusing on their content of iron and iron absorption inhibitors, like polyphenols and phytic acid, guided the development of a nutritional formula rich in bioavailable iron. In addition, their antioxidant potential was assessed.

Furthermore, this study involved two in vivo experiments on rats with IDA. One aspect of the study assessed the formula’s effect on iron bioavailability when administered alongside iron supplementation, aiming to evaluate whether the formula has any impact on the efficacy of iron supplementation. The other examined iron bioavailability when the formula was consumed alone. Moreover, despite the established knowledge that polyphenols and phytic acid diminish iron bioavailability, the precise quantities of these compounds alongside iron that support iron bioavailability have remained undisclosed until now.

## 2. Materials and Methods

### 2.1. Development of Nutritional Formula

#### 2.1.1. Literature Review

The ultimate goal of this study was to develop an innovative nutritional formula that could serve as a versatile base for creating various nutraceutical products for enhancing blood iron level and improving other main blood IDA parameters. The concept of development of the nutritional formula was based on two key principles: (1) the nutraceutical prepared according to the formula must contain a certain amount of the plant-based foods that naturally contain high iron content; (2) nutraceutical composition must not include foods containing compounds that inhibit iron absorption, or it may contain them in trace amounts. The compounds proven to inhibit iron absorption are certain plant polyphenols and phytic acid.

The first step in the development of a nutrition formula was an in-depth literature review in order to include or exclude individual plant foods from examination. The literature review was conducted in autumn 2020 and included a review of different electronic databases including the following: SCOPUS (https://www.scopus.com, accessed on 27 October 2025), PubMed (https://pubmed.ncbi.nlm.nih.gov, accessed on 27 October 2025), USDA Database for the Flavonoid Content of Selected Foods. Release 3.2 [36], Phenol-Explorer (http://phenol-explorer.eu, accessed on 27 October 2025) [37], and USDA National Nutrient Database for Standard Reference Legacy [38].

Based on the data in the literature, individual plant foods that naturally contain a high content of iron and at the same time do not contain compounds that inhibit iron absorption, i.e., certain plant polyphenols and phytic acid, or contain them in trace amounts, were identified and included in the further study. Also, individual plant foods that contain high amounts of compounds proven to inhibit iron absorption were identified and excluded from the study. Specifically, compounds whose presence in plant foods served as exclusion criteria were flavonoids that strongly chelate iron, such as flavonoids that have one of the combinations of functional groups: 3-hydroxy- and 4-carboxyl group, 5-hydroxy and 4-carboxyl group, or 3′- and 4′-hydroxyl groups (i.e., quercetin, genistein, luteolin, apigenin, epigallocatechin-3-gallate, and their glycosides, etc.). Also, the presence of phytic acid as well as phenolic acids, which have two hydroxyl groups in the ortho position or a hydroxyl and a carboxyl group in the ortho position, were used as exclusion criteria.

According to the systematic literature review regarding the presence of iron and iron absorption inhibitors, as well as their availability on the markets worldwide and the feasibility of processing them in industrial settings, eight plant foods were identified as those with a preferable chemical profile and included in the study. The chosen plant-based foods were white potato (boiled, WPB), beetroot (cold-pressed juice, BEE), kiwi (cold-pressed juice, KIW), pineapple (cold-pressed juice, PIN), butternut squash (boiled, BSB), melon (cold-pressed juice, MEL), cinnamon powder (CIN), and acacia honey (HON).

#### 2.1.2. Preparation of Plant Foodstuffs and Nutraceuticals

All chosen plant foods were purchased from different markets in Novi Sad, Serbia. The samples were collected randomly in sufficient quantities (at least 2 kg) to obtain representative samples. The samples were processed in the following ways: WPB and BSB were peeled and chopped in cubed slices, which were boiled in water for 30 min, and then the water was removed. BEE, KIW, PIN and MEL were peeled and cold-pressed using an electronic juicer to obtain juices.

Afterwards, WPB, BSB, BEE, KIW, PIN, MEL, CIN, and HON were analysed for their total phenols, flavonoids, phytic acid and iron contents, and qualitative and quantitative polyphenol profiles by HPLC-MS/MS. Afterwards, two different nutraceuticals (smoothies) were made by mixing exact amounts of plant foods and homogenisation was performed using an electric blender.

The first nutraceutical, named orange smoothie (ORG), was made by mixing 60 g of WPB, 100 g of BSB, 60 mL of PIN, 1 g of CIN, 5 g of HON, and 70 mL of H_2_O, which was 300 mL in total and was marked as one serving.

The second nutraceutical, named red smoothie (RED), was made by mixing 100 g of WPB, 100 mL of MEL, 70 mL of KIW, 5 mL of BEE, and 5 g of HON, which was 300 mL in total and was marked as one serving.

ORG and RED were made without adding artificial components. The smoothies had a characteristic sweet-sour flavour, which was informally reported as pleasant by individuals involved in their preparation. However, no formal sensory evaluation was conducted. ORG and RED were analysed for their total phenols, flavonoids, phytic acid and iron contents, and qualitative and quantitative polyphenol profile by HPLC-DAD-MS/MS. Afterwards, in order to examine the efficacy of ORG and RED in enhancing blood iron levels and improving other main blood IDA parameters when consumed, two sets of in vivo experiments on animals were carried out.

### 2.2. Chemical Analysis of Plant-Based Foodstuffs and Nutraceuticals

#### 2.2.1. Phenolic Profile Determination

The phenolic profile of eight plant-based foodstuffs and two nutraceuticals was determined by evaluation of total phenolic and flavonoid contents and HPLC-DAD-MS/MS analysis. Prior to the analysis, all samples except CIN and HON, were dehydrated in vacuo at 45 °C. The dried residues, as well as CIN and HON, were further extracted with 70% of aqueous methanol, 15 min in an ultrasonic bath, followed by 1 h on a shaker (120 rpm/min), whereby both were at room temperature. The extracts of each sample were prepared in triplicate.

Total phenolic and flavonoid contents were determined according to spectrophotometric methods described previously [39].

Furthermore, filtered samples were used for qualitative and quantitative analysis of phenolic compounds using high-performance liquid chromatography (HPLC—Agilent Technologies 1200 Series) coupled with UV/VIS and tandem mass spectrometry with electrospray ionisation (ESI-QqQ-MS/MS—Agilent Technologies 6410A). Briefly, 5 µL of the sample was injected into the system, and compounds were separated on a Zorbax Eclipse XDB-C18 (50 mm × 4.6 mm, 1.8 µm) rapid resolution column (Agilent Technologies, Santa Clara, CA, USA) held at 50 °C. Mobile phase consisting of 0.05% of aqueous formic acid (A) and methanol (B) was delivered at a flow rate of 0.5 mL/min in gradient mode (0 min 30% B; 12 min 70%; 18 min 100%; 24 min 100%; re-equilibration time was 6 min). Eluted compounds were monitored at 340 nm in order to detect potential inhibitors of iron absorption, since flavonoids and most of the phenylpropanoids absorb light at that wavelength. For quantification of the 44 selected phenolic compounds, data were acquired in dynamic MRM mode, using the optimised compound-specific parameters (retention time, precursor ion, product ion, fragmentor voltage, and collision voltage) [40]. For all the compounds, peak areas were determined using Agilent MassHunter Workstation Software—Qualitative Analysis (ver. B.06.01). Calibration curves were plotted and samples’ concentrations were calculated using the OriginLabs Origin Pro (ver. 8.0) software.

#### 2.2.2. Total Phytic Acid Content

Pythic acid content was measured using a commercial kit according to manufacturer’s instructions (Phytic Acid Assay Kit, Megazyme, Wicklow, Ireland).

#### 2.2.3. Total Iron Content

Total iron content was examined by atomic absorption spectrophotometry. Before the analysis, all samples, except CIN and HON, were dehydrated in vacuo at 45 °C and the dry residue, or CIN and HON (approx. 1 g), was digested with 10 mL of ccHNO_3_ and 2 mL of ccH_2_O_2_, on a temperature of 180 °C, until the full mineralisation. After filtration and dilution with deionised H_2_O, samples were analysed on an atomic absorption spectrometer (Perkin Elmer, Waltham, MA, USA, Atomic Absorption Spectrometer, AAnalyst 700”) using flame emission spectrometry in accordance with the standard method EPA 7000B. The practical limit of quantitation for iron content in samples was 0.011 mg/L. Measurements were performed in triplicate.

### 2.3. Antioxidant Potential of Plant-Based Foodstuffs and Nutraceuticals

#### 2.3.1. DPPH Assay

The antioxidant activity was evaluated by DPPH assay according to the method described by Lesjak et al. [39]. Briefly, 10 µL of the extract was mixed with 190 µL of MeOH and 100 µL of 67.5 µM DPPH solution in MeOH. Trolox was used for the construction of the standard curve (0.16–1 mM in DMSO) and results are expressed as mM of Trolox equivalents per g of plant food or mL of juice/smoothie.

#### 2.3.2. FRAP Assay

FRAP assay was performed according to the method described by Lesjak et al. [39]. Briefly, 10 µL of the extract was mixed with 290 µL of the FRAP reagent. Trolox was used for the construction of the standard curve (0.0005–1 mM) and results are expressed as mM of Trolox equivalents per g of plant food or mL of juice/smoothie.

### 2.4. Experimental Animals

All experimental procedures in animals were approved by the Ethics Committee of the University of Novi Sad, Republic of Serbia (approval No. EK: IV-E-2020-02) and were conducted in accordance with the European Commission legislation on the protection of animals used for scientific purposes, Directive 2010/63/EU, and the Law on Animal Welfare of Republic of Serbia [41,42]. In addition, during the entire research, the 3R principles were applied.

Male Wistar rats (three weeks old) were supplied by the breeding centre from the Military Medical Academy, Belgrade, Republic of Serbia. The animals were kept in the vivarium at the Department of Pharmacology and Toxicology, Faculty of Medicine University of Novi Sad, Novi Sad, Republic of Serbia, and appropriate conditions were applied (temperature of 25 °C, relative air humidity of 30–50%, and daily lighting cycle, i.e., 12 h of light/12 h of darkness). Animals were placed in collective cages (six individuals per cage).

#### 2.4.1. In Vivo Study on the Effects of the Nutraceuticals on Iron Bioavailability from Oral Iron Supplementation When Applied Simultaneously

**Figure 1:**
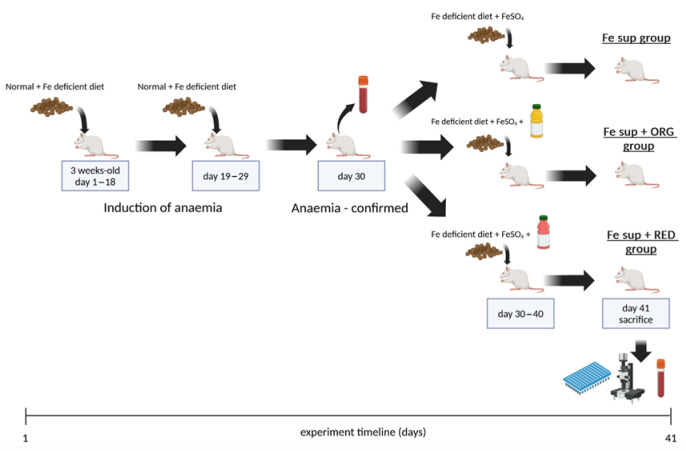
Timeline of experiment which examined effects of nutraceuticals ORG and RED on the in vivo iron bioavailability from oral iron supplementation when applied simultaneously. The figure was created with BioRender.

The study was divided into two stages. The aim of the first stage was to achieve IDA in all animals by dietary intervention. During the second stage, animals were simultaneously orally treated (known as oral gavage) with nutraceuticals ORG and RED and supplemented with iron. The timeline of the experiment is shown in Figure 1.

During the first stage, three weeks old rats were placed on a special diet in order to cause IDA in rats. A special diet was applied for 29 consecutive days following this regimen: from day 1 to day 18 (a total of 18 days), the animals were fed with a mixed diet which was 50:50 in composition, consisting of a diet containing optimum iron content, i.e., 270 mg Fe/kg (diet type laboratory rats 20%, manufacturer Veterinarski zavod Subotica, Stočna hrana doo, Subotica, Republic of Serbia) and a diet with low iron content (5.9 mg Fe/kg) (type iron deficient 5.9 mg/kg Fe, U8958P Version 0176, manufacturer SAFE, Augy, France); from day 19 to day 29 (a total of 11 days), the animals were fed only with rat diet with low iron content containing 5.9 mg Fe/kg. During this stage, water and food were supplied ad libitum. On day 30, blood was taken from the tail vein and IDA was confirmed by haemoglobin level (<13.7 g/dL). Additionally, analysis of blood samples was performed as explained in the text below.

The second stage of the experiment began right after IDA was confirmed on day 30. Animals were divided into three experimental groups and treated accordingly:

Fe sup (control group; 12 animals at the start; 10 animals at the end of the experiment) was treated once a day orally by gastric tube with a 160 µL mixture solution of FeSO_4_ and ascorbic acid (one dose contained 1.24 mg Fe and 6.576 mg ascorbic acid). Mixture solution was prepared as follows: 1.991 g of FeSO_4_ × 7H_2_O and 2.055 g of ascorbic acid dissolved in 50 mL of saline (0.9% *w*/*v* of NaCl).

ORG + Fe sup (experimental group ORG + Fe sup; twelve animals at the start; nine animals at the end of the experiment) was treated twice a day orally by gastric tube with ORG supplemented with FeSO_4_ and ascorbic acid. The animals were treated in two doses; the first dose was 1.3 mL and the second dose was 1.26 mL (a total of 2.56 mL per day), with an interval of 3 h between doses. FeSO_4_ × 7H_2_O and ascorbic acid were added to the ORG, so that the daily dose of 2.56 mL contained 1.24 mg of Fe and 6.576 mg of ascorbic acid.

RED + Fe sup (experimental group RED + Fe sup; twelve animals at the start; seven animals at the end of the experiment) was treated twice a day orally by gastric tube with RED supplemented with FeSO_4_ and ascorbic acid. The animals were treated in two doses; first dose was 1.3 mL and second dose was 1.26 mL (a total of 2.56 mL per day), with an interval of 3 h between doses. FeSO_4_ × 7H_2_O and ascorbic acid were added to the RED, so that the daily dose of 2.56 mL contained 1.24 mg of Fe and 6.576 mg of ascorbic acid.

Described treatments in the second stage lasted 11 consecutive days. During the treatment, the animals were fed only with rat diet with low iron content containing 5.9 mg Fe/kg. Throughout the experiments, rats were afforded unrestricted access to water, while their dietary intake was meticulously regulated. Specifically, food provision was suspended on a nightly basis to facilitate gastric emptiness for the subsequent administration of two oral treatments in the morning. Following the treatment, food was reinstated to the rats, who underwent a total dietary restriction of approximately 15 h. Subsequently, rats were granted unimpeded access to food until the subsequent evening period.

Sacrifice was performed on the 12th day of the second stage. Following administration of a terminal intraperitoneal injection (IP) dose of pentobarbitone sodium (120 mg/kg body weight), a blood sample was removed via cardiac puncture and used for further analysis. Additionally, the duodenum, liver, spleen, and kidney were removed, and two samples from each organ were taken. First one was taken for histological analysis; the second one was rapidly frozen in liquid nitrogen before being stored at −80 °C and subsequently used for determination of tissue non-haem iron content and gene expression levels.

#### 2.4.2. In Vivo Study on the Effects of the Nutraceutical ORG on Iron Bioavailability

**Figure 2:**
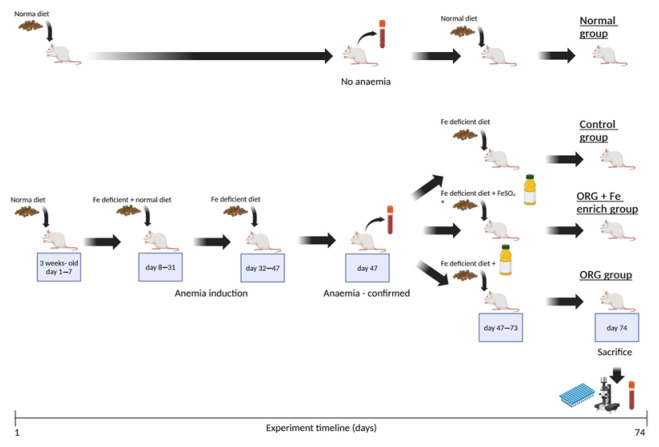
Timeline of the experiment which examined the effects of nutraceutical ORG on the in vivo iron bioavailability. The figure was created with BioRender.

The study was divided in two stages. The aim of the first stage was to achieve IDA in three groups of animals (Control, ORG, ORG + Fe-enriched). During the second stage, one group (Control) of animals was not treated and used as a control, the second (ORG) group was treated only with ORG, and the third group (ORG + Fe-enriched) was treated with ORG enriched with iron. During the second stage, the animals were orally treated (oral gavage) with the treatment corresponding to their respective group. In parallel, during both stages of the study, a separate group of animals (Normal) was fed with a diet containing optimum iron content (270 mg Fe/kg) and was not treated. This group was formed for comparison purposes. The timeline of the experiment is shown in Figure 2.

During the first stage, three-week-old rats were placed on a special diet in order to cause IDA. a special diet was applied for 47 consecutive days following this regimen: from day 1 to day 7 (a total of 7 days), the animals were fed only with the diet containing optimum iron content (270 mg Fe/kg); from day 8 to day 31 (a total of 24 days), the animals were fed with a mixed diet with a 50:50 composition, with a diet containing optimum iron content (270 mg Fe/kg) and a diet with a low iron content of 5.9 mg Fe/k; from day 32 to day 47 (a total of 16 days), the animals were fed only with rat diet containing 5.9 mg Fe/kg. During this stage, water and food were supplied ad libitum. On day 47, blood was taken from the tail vein and IDA was confirmed by haemoglobin level (<13.7 g/dL). Additionally, analysis of blood samples was performed as explained in the text below.

The second stage of the experiment began on day 47 right after IDA was confirmed in the animals in the groups (Control, ORG, and ORG + Fe-enriched).

The animals were divided into four experimental groups and treated accordingly:

Control (the control group; 11 animals at the start and at the end of the experiment) had no treatment at all but was fed only with a diet with low iron content of 5.9 mg Fe/kg.

Normal (the control group; six animals at the start and at the end of the experiment) had no treatment at all and was fed with a diet containing optimum iron content for feeding rats (270 mg Fe/kg).

ORG (experimental group ORG; ten animals at the start; nine animals at the end of the experiment) was treated once a day orally by gastric tube with 1.2 mL of concentrated ORG enriched with ascorbic acid. Ascorbic acid was added to the ORG, so that the daily dose of 1.2 mL contained 0.58 mg of ascorbic acid. Compared to ORG in the previous experiment, this concentrated ORG had 24% less water, so the dose of 1.2 mL in this experiment corresponds to the dose of 1.56 mL in the previous experiment. The reduction in water and overall treatment volume in ORG, compared to the first treatment, was implemented to reduce mortality in rats caused by aspiration pneumonia from inhaling stomach contents.

ORG + Fe-enriched (experimental group ORG + Fe-enriched; 10 animals at the start and at the end of the experiment) was treated once a day orally by gastric tube with 1.2 mL of concentrated ORG enriched with Fe and ascorbic acid. FeSO_4_ × 7H_2_O and ascorbic acid were added to the ORG, so that the daily dose of 1.2 mL contained 0.11 mg of Fe and 0.58 mg of ascorbic acid.

Described treatments in the second stage lasted 27 days. During treatments, the animals in the groups (Control, ORG, and ORG + Fe-enriched) were fed only with diet with low iron content containing 5.9 mg Fe/kg. During this stage, water and food were supplied ad libitum.

Sacrifice was performed the day after the end of the second stage. Following administration of a terminal IP dose of pentobarbitone sodium (120 mg/kg body weight), a blood sample was removed via cardiac puncture and used for further analysis. Additionally, the duodenum, liver, spleen, and kidney were removed, and two samples from each organ were taken. The first one was taken for histological analysis; the second one was rapidly frozen in liquid nitrogen before being stored at −80 °C, and subsequently used for determination of tissue non-haem iron content and gene expression levels.

### 2.5. Analysis of Blood Samples

Blood sampling was performed on two occasions during each study. The first blood sampling was performed from the tail vein after introduction of rats into IDA (before the second stage treatments). The second blood sampling was performed during cardiac puncture, after the second stage treatments.

For biochemical analysis, blood was collected into microtainers (from tail vein) or vacutainers (during cardiac puncture), whereby both were filled with separating gels and a clot activator. Serum was separated from the clotted blood after centrifugation (15 min at 3000× *g*, room temperature) and 30 min after sampling. For haematological analyses, blood was collected into EDTA microtainers (from tail vain and during cardiac puncture).

Serum iron content and unsaturated iron-binding capacity (UIBC) were determined on an automatic biochemical analyser A25 (Biosystems, Barcelona, Spain).

Serum iron was determined by the spectrophotometric method. In brief, Fe^3+^ was liberated from transferrin by guanidine, followed by the reduction of Fe^3+^ to Fe^2+^ by ascorbate. Reduced iron reacted with ferrozine to form a coloured complex whose concentration was determined spectrophotometrically.

UIBC was determined by adding a known amount of Fe^2+^ to the samples, while the excess Fe^2+^ reacted with ferrozine to form a coloured complex whose concentration was determined.

Total iron-binding capacity (TIBC) was determined by summing values of serum iron and UIBC. The percentage of transferrin saturation (TS) with iron is calculated by dividing the serum iron concentration by TIBC and multiplying by 100.

Haematological parameters (red blood cells (RBCs), haemoglobin (Hgb), red cell distribution width (RDW), mean corpuscular volume (MCV), mean corpuscular haemoglobin (MCH), mean corpuscular haemoglobin concentration (MCHC), haematocrit (HCT), platelet count (PLT), white blood cell (WBC), and number/percentage of granulocytes, monocytes and lymphocytes) were determined on an automatic haematology analyser BC-2800 Vet (Mindray, Shenzhen, China).

The concentrations of ferritin and transferrin were determined using commercial ELISA tests (Elabscience, Houston, TX, USA).

### 2.6. Histopathological Analysis

The histological assessment was blind, performed by two researchers by light microscopy. The histological analysis was performed on a sample of liver, spleen, kidney, and duodenal tissue sampled from each animal. Samples were fixed in Bouin’s solution for 24 h. After that, samples were dehydrated in a graded series of isopropyl alcohol and embedded in paraffin blocks. For each sampled organ, 5 µm thick tissue sections were cut, using a rotation microtome (Sakura Finetek USA, Inc., Torrance, CA, USA). Sections were stained with routine hematoxylin and eosin method (H&E), in addition to the Prussian blue staining method applied to spleen tissue sections. All histological slides were analysed under a light microscope (Leica DM LB, Wetzlar, Germany) and photographed using integrated camera (Leica DC 100).

### 2.7. Non-Haem Tissue Iron

Quantitative analysis of non-haem iron was carried out according to the method of Torrance and Bothwell [43]. Briefly, 20–50 mg of tissue sample (duodenum, liver, spleen, and kidney) was dried at 55 °C for 72 h and subsequently weighed. Dried samples were digested in 1 mL of acid mixture (30% (*v*/*v*) HCl and 10% (*w*/*v*) trichloroacetic acid) at 65 °C for 20 h. A total of 1 mL of chromogen reagent (0.1% (*w*/*v*) bathophenanthrolinesulfonate; 1% (*v*/*v*) thioglycolic acid in 50% (*w*/*v*) sodium acetate solution) and samples (supernatant of digestate) were incubated at 37 °C for 10 min, after which the absorbance of samples and iron standards was measured at 535 nm. Results were calculated as µg iron/g of dry tissue weight (dw).

### 2.8. RNA Extraction and RT-PCR

The gene expression of proteins of interest for iron homeostasis, such as Dcytb, DMT1, hephaestin, FPN, ferritin light chain, hepcidin, and GAPDH, were measured according to the procedure explained below.

Total RNA from tissues (duodenum, liver, spleen, and kidney) was extracted with the TRIzol^®^ reagent (Thermo Fisher Scientific, Waltham, MA, USA), according to the manufacturer’s instructions. The concentration of extracted RNA was measured using a Qubit^®^ 2 fluorometer and Qubit RNA BR Assay Kit according to manufacturer’s instructions (Thermo Fisher Scientific Inc.).

Afterwards, total RNA (1 µg) was treated with recombinant deoxyribonuclease (DNase I, Thermo Fisher Scientific Inc.) and reverse transcribed using the High-capacity cDNA Reverse Transcription Kit (Applied Biosystems, Waltham, MA, USA), according to the manufacturer’s instructions.

RT-PCR reactions were performed using a M×3005P (Stratagene, La Jolla, CA, USA) and iTaq Universal SYBR Green Supermix kit (Bio Rad, Hercules, CA, USA), according to the manufacturer’s instructions. Each reaction was performed in duplicate. Samples without cDNA were included as negative controls. The primer sequences used for each gene are given in Supplementary Materials Table S7. Quantitative measurements of iron transporter relative to GAPDH gene expression were derived using the ΔCt method.

### 2.9. Statistics

All data are presented as mean ± standard error of the mean (SEM). The statistically significant difference between groups was determined using Student’s two-tailed unpaired *t*-test. Statistical significance was taken to be *p* ≤ 0.05.

## 3. Results

### 3.1. Design of Nutritional Formula and Nutraceuticals

Firstly, individual plant samples, and afterwards ORG and RED, were analysed for their total phenols, flavonoids, phytic acid and iron contents, and qualitative and quantitative polyphenol profile by HPLC-DAD-MS/MS.

HPLC-MS/MS analysis revealed that most of the tested individual polyphenols, especially those marked as potent iron chelators (e.g., quercetin, myricetin, catechins, chlorogenic acid, etc.), were not present in analysed samples or were detected in traces, such as in KIW (Table 1). The only exception was the CIN sample. However, the content of CIN in the nutraceutical ORG was low (1 g CIN/300 mL ORG), thus the concentration of polyphenols detected in ORG was also low.

**Table 1:** Content ^a^ of quantified polyphenols in selected plant-based foodstuffs and nutraceuticals (ORG and RED).

Compound	Plant-Based Foodstuffs								Nutraceuticals	
	WPB ng/g	BEE ng/mL	KIW ng/mL	PIN ng/mL	BSB ng/g	MEL ng/mL	CIN ng/g	HON ng/g	ORG ng/mL	RED ng/mL
Phenolic acids										
*p*-Hydroxybenzoic acid	<LoQ ^b^	<LoQ	<LoQ	<LoQ	<LoQ	<LoQ	12,808.04 ± 514.25	3645.60 ± 177.32	123.33 ± 5.56 a ^c^	41.38 ± 2.78 b
Cinnamic acid	<LoQ	<LoQ	<LoQ	<LoQ	<LoQ	<LoQ	494,341.61 ± 2833.43	<LoQ	1597.42 ± 47.47	<LoQ
Protocatechuic acid	14.93 ± 0.38	<LoQ	18.99 ± 1.74	<LoQ	11.68 ± 0.52	<LoQ	137,959.64 ± 866.64	568.76 ± 28.32	461.21 ± 42.83 a	54.44 ± 0.60 b
Gentisic acid	<LoQ	<LoQ	<LoQ	<LoQ	<LoQ	<LoQ	371.35 ± 5.13	<LoQ	<LoQ	<LoQ
*p*-Coumaric acid	<LoQ	12.56 ± 1.07	7.97 ± 0.74	<LoQ	13.87 ± 0.98	33.81 ± 0.77	2778.11 ± 289.13	944.87 ± 36.11	179.50 ± 9.47 a	28.67 ± 2.07 b
*o*-Coumaric acid	<LoQ	<LoQ	<LoQ	<LoQ	<LoQ	<LoQ	9208.65 ± 675.49	<LoQ	21.89 ± 1.55	<LoQ
Gallic acid	<LoQ	<LoQ	<LoQ	27.63 ± 1.52	<LoQ	<LoQ	2734.83 ± 125.74	382.64 ± 32.86	26.84 ± 2.25 a	23.41 ± 1.12 b
Vanillic acid	<LoQ	<LoQ	<LoQ	<LoQ	<LoQ	222.23 ± 13.83	5394.00 ± 478.74	<LoQ	<LoQ	168.82 ± 11.76
Caffeic acid	95.06 ± 3.78	17.42 ± 1.58	73.42 ± 6.78	<LoQ	<LoQ	<LoQ	319.40 ± 25.24	950.55 ± 48.29	355.73 ± 32.10 b	1427.64 ± 52.11 a
3,4-Dimethoxycinnamic acid	<LoQ	<LoQ	<LoQ	<LoQ	<LoQ	<LoQ	<LoQ	2156.78 ± 134.52	<LoQ	<LoQ
Ferulic acid	90.98 ± 6.33	4638.78 ± 383.12	<LoQ	<LoQ	<LoQ	<LoQ	3285.53 ± 140.67	<LoQ	118.69 ± 8.44 a	122.39 ± 11.13 a
Syringic acid	<LoQ	976.67 ± 57.76	<LoQ	<LoQ	<LoQ	<LoQ	6745.86 ± 334.56	<LoQ	45.76 ± 1.87 b	50.13 ± 2.48 a
5-*O*-caffeoylquinic acid (chlorogenic acid)	10,075.24 ± 831.08	43.58 ± 3.18	47.76 ± 3.69	27.97 ± 2.12	59.32 ± 2.52	<LoQ	1663.97 ± 45.88	333.01 ± 22.21	4679.01 ± 62.64 a	10,812.91 ± 105.22 b
Flavonoids										
Catechin	<LoQ	<LoQ	<LoQ	<LoQ	<LoQ	<LoQ	49,249.49 ± 2589.33	<LoQ	<LoQ	<LoQ
Epicatechin	<LoQ	<LoQ	2537.28 ± 218.30	<LoQ	<LoQ	<LoQ	16,008.53 ± 136.99	<LoQ	<LoQ	239.62 ±17.20
Kaempferol 3-*O*-glucoside	<LoQ	<LoQ	82.29 ± 7.33	43.54 ± 2.33	<LoQ	<LoQ	749.08 ± 6.31	<LoQ	7.02 ± 0.61 a	20.72 ± 0.72 b
Chrysoeriol	<LoQ	<LoQ	<LoQ	<LoQ	<LoQ	<LoQ	<LoQ	<LoQ	210.74 ± 2.07	<LoQ
Quercetin 3-*O*-glucoside	<LoQ	<LoQ	<LoQ	<LoQ	<LoQ	<LoQ	617.85 ± 21.99	<LoQ	<LoQ	33.38 ± 0.39
Quercetin 3-*O*-galactoside	<LoQ	<LoQ	133.42 ± 11.76	<LoQ	<LoQ	<LoQ	<LoQ	<LoQ	<LoQ	<LoQ
Rutin	14.13 ± 1.12	<LoQ	265.62 ± 18.76	<LoQ	<LoQ	<LoQ	153.81 ± 11.52	127.53 ± 10.64	302.19 ± 4.14 a	659.78 ± 12.44 a
Apigenin 3-*O*-glucoside	<LoQ	<LoQ	<LoQ	<LoQ	<LoQ	<LoQ	91.15 ± 7.67	<LoQ	<LoQ	<LoQ
Appin	<LoQ	<LoQ	<LoQ	<LoQ	<LoQ	<LoQ	84.89 ± 7.58	<LoQ	<LoQ	<LoQ
Quercitrin	<LoQ	<LoQ	996.26 ± 38.76	<LoQ	<LoQ	<LoQ	4611.08 ± 34.56	<LoQ	14.66 ± 1.66 a	344.16 ± 3.02 b
Naringenin	<LoQ	<LoQ	<LoQ	<LoQ	<LoQ	<LoQ	599.08 ± 21.98	147.75 ± 9.35	<LoQ	<LoQ
Coumarines										
Umbelliferone	<LoQ	<LoQ	<LoQ	<LoQ	<LoQ	<LoQ	139.23 ± 2.21	<LoQ	<LoQ	<LoQ
Skopoletin	<LoQ	<LoQ	<LoQ	<LoQ	<LoQ	<LoQ	397.81 ± 31.44	<LoQ	<LoQ	32.29 ± 2.41
Aesculetin	<LoQ	<LoQ	<LoQ	<LoQ	<LoQ	<LoQ	<LoQ	146.56 ± 9.45	<LoQ	77.01 ± 4.38
Lignane										
Secoisolariciresinol	<LoQ	<LoQ	<LoQ	<LoQ	<LoQ	<LoQ	4468.53 ± 256.44	<LoQ	<LoQ	<LoQ

^a^ Values are means ± SEM of three measurements. ^b^ Below instrument quantification limit (LoQ). ^c^. Means within each row with different letters (a–b) differ significantly (*p* ≤ 0.05). Abbreviations: BEE: beetroot cold-pressed juice; BSB: butternut squash boiled; CIN: cinnamon powder; HON: acacia honey; KIW: kiwi cold-pressed juice; MEL: melon cold-pressed juice; ORG: nutraceutical, named orange smoothie; PIN: pineapple cold-pressed juice; RED: nutraceutical, named red smoothie; WPB: white potato boiled. Analysed but not detected: amentoflavone, apigenin, baicalein, baicalin, daidzein, epigallocatechin gallate, genistein, isorhamnetin, kaempferol, luteolin, luteolin 7-*O*-glucoside, matairesinol, myricetin, quercetin, and vitexin.

Through HPLC-MS/MS analysis of the two nutraceuticals, it was noted that RED contained 1.7 times more polyphenols than ORG, with the most notable difference observed in the levels of phenolic acids. Most of the detected phenolic acids were marked as weak inhibitors of iron absorption (Table 1).

Samples exhibited low levels of total phenolic and flavonoid contents, except for CIN (Table 2). Moreover, the HPLC-DAD chromatograms of plant-based foodstuffs showed a minimal number of small peaks, consistent with the findings of the total flavonoid content assay. Only in the CIN sample, some higher peaks were found (Figure S1). All analysed foodstuffs contained a certain amount of iron, especially CIN and WPB. All examined foodstuffs had low levels of phytic acid, with the exception of CIN and HON, which contained more than 2 mg of phytic acid per g (Table 2).

Antioxidant activity of the samples was evaluated by DPPH and FRAP assays. Nutraceutical RED showed stronger antioxidant activity compared to nutraceutical ORG. In ORG formulation, the primary contributor to antioxidant capacity was PIN; whereas in RED, KIW and MEL were the dominant contributors. Although CIN demonstrated the highest antioxidant activity among all individual ingredients, consistent with its high phenolic content compared to other plant-based foodstuffs, it was included in ORG in only a small amount. Similarly, despite the high antioxidant potential of RED, its most active component BEE was also present in low concentration.

### 3.2. Effects of Nutraceuticals on the In Vivo Iron Bioavailability from Oral Iron Supplementation When Applied Simultaneously

**Figure 3:**
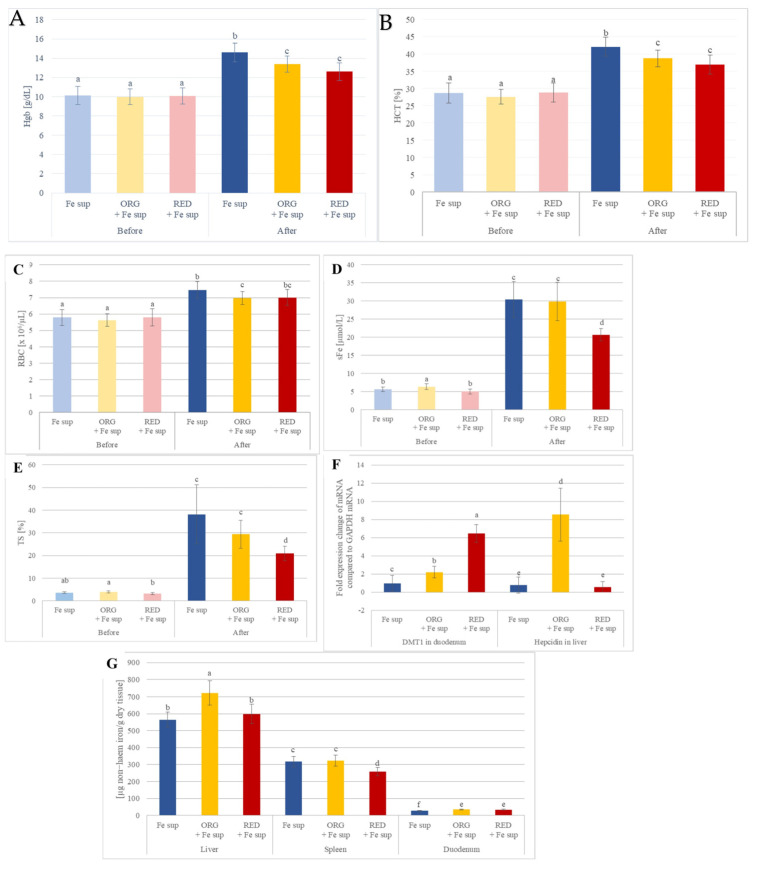
Selected blood parameters, tissue iron levels, and estimation of gene expression of proteins of interest for iron homeostasis of rats with IDA that were simultaneously orally treated with nutraceuticals ORG or RED and supplemented with iron: (**A**) haemoglobin (Hgb); (**B**) haematocrit (HCT); (**C**) red blood cells (RBCs); (**D**) serum iron (sFe); (**E**) transferrin saturation (TS); (**F**) gene expression. In groups ORG + Fe sup and RED + Fe sup, expression of DMT1 mRNA was significantly increased by 2.2 and 6.6 times, respectively, when compared with the Fe sup group. This occurrence was probably due to IDA in the rats 55. Hepcidin mRNA in liver was greatly increased in ORG + Fe sup compared with all other groups from this study (10.9 times when compared with Fe sup, and 14.7 times when compared with RED + Fe sup). (**G**) Tissue iron level values are means ± SEM of three measurements. Means with different letters (a−f) differ significantly (*p* ≤ 0.05).

**Figure 4:**
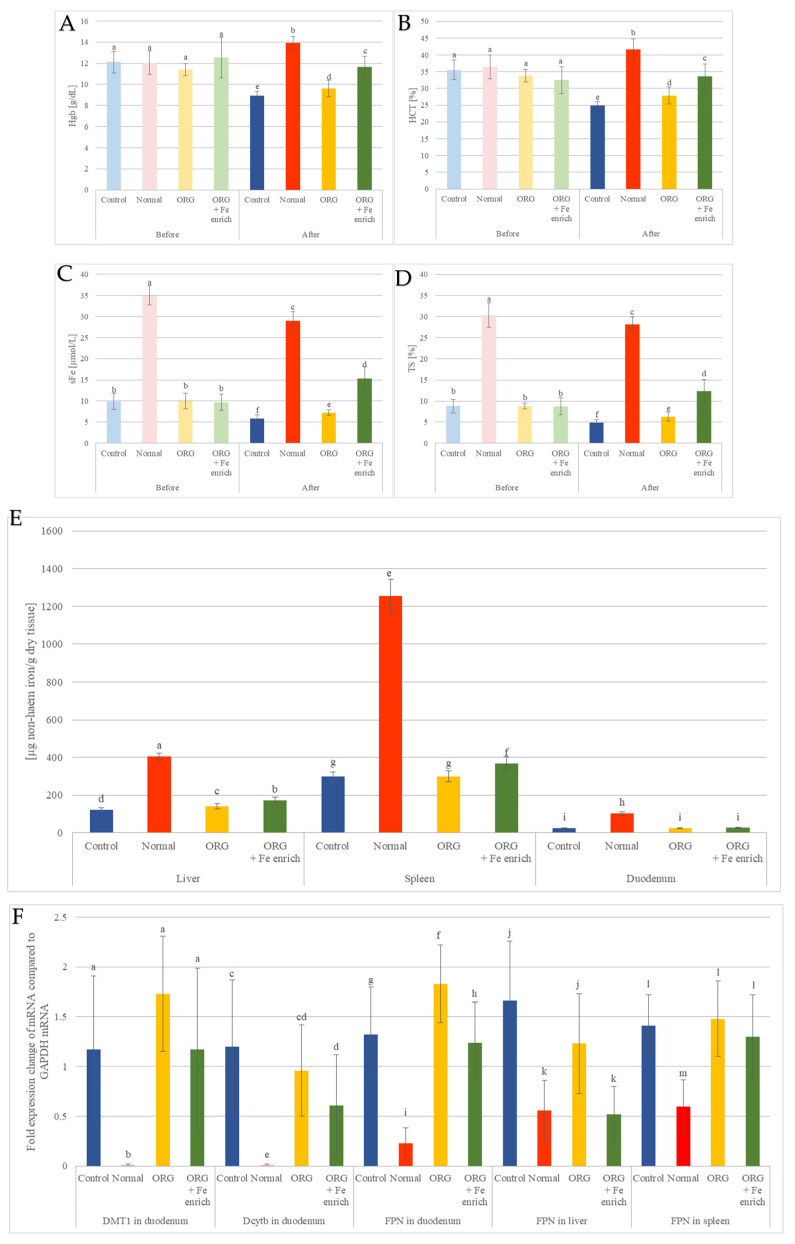
Selected blood parameters, tissue iron levels and estimation of gene expression of proteins of interest for iron homeostasis of rats with IDA that were treated with nutraceutical ORG: (**A**) haemoglobin (Hgb); (**B**) haematocrit (HCT); (**C**) serum iron (sFe); (**D**) transferrin saturation (TS); (**E**) gene expression. In the control group, ORG, and ORG + Fe-enriched groups, the expression of DMT1 and Dcytb mRNA in the duodenum was significantly elevated, surpassing 100 and 60 times, respectively, compared to the normal group. Additionally, FPN mRNA levels in the duodenum, liver, and spleen were significantly higher in the control group, ORG, and ORG + Fe-enriched groups, exceeding 5.4, 3, and 2.1 times, respectively, in comparison to the normal group. (**F**) Tissue iron levels values are means ± SEM of three measurements. Means with different letters (a–m) differ significantly (*p* ≤ 0.05).

Before the treatment, severe IDA was confirmed in all animal groups by low Hgb, HCT, RBC, TS, serum iron, and transferrin levels (Figure 3 and Figure 4, Table S1) [44,45].

Initially, all experimental groups displayed elevated WBC counts, which are not typically characteristic of IDA. Instead, such elevations are more commonly associated with underlying or concurrent inflammatory responses. This inflammatory component may have been triggered by several factors, including physiological stress related to the induction of anaemia, dietary interventions, or handling and repeated gavage procedures. Inflammatory responses can activate immune cells and lead to transient increases in leukocyte populations, particularly monocytes and granulocytes, which are frontline responders in innate immunity. Importantly, we observed that WBC levels decreased by the end of the 11-day treatment period, suggesting a resolution or reduction in the initial inflammatory response. This trend supports the idea that the initial leukocytosis was transient and possibly linked to the induction or early phase of the experimental protocol rather than the anaemia itself.

Additionally, the presence of inflammation can affect the interpretation of certain iron-related markers, particularly hepcidin and serum ferritin, both of which are positive acute-phase reactants. Due to this well-recognised confounding effect, we did not use hepcidin or ferritin as primary indicators of iron status in this model, which is in accordance with current recommendations [44,45].

After the treatment, all animal groups showed improvement in the main blood IDA parameters. The most notable increases were in TS and serum iron levels, which were 8.1 and 4.8 times higher, respectively, than at the start of the experiment. Hgb, HCT, and RBC levels increased by 1.2 to 1.5 times (Figure 3, Table S1).

The comprehensive assessment of histological characteristics in the duodenum, liver, spleen, and kidney revealed tissue changes consistent with improved iron status following the concurrent administration of ORG and RED alongside iron supplementation (Figures S2–S5).

Liver and spleen iron pools were found to be the highest in the ORG + Fe sup group, which was confirmed by histological analysis where the iron depot was increased. Levels of duodenal iron were highest and the same in groups where ORG and RED were administered simultaneously with iron supplementation (Figure 3, Table S2).

It is evident that gene expression was somewhat consistent in all study groups, except for the expression of mRNA for DMT1 in the duodenum and hepcidin in the liver. This occurrence could be explained by high iron in the liver of animals from the ORG + Fe sup group, since high iron could be a signal for iron overload and call for hepcidin expression, primarily by liver (Figure 3, Table S3).

### 3.3. Effects of Nutraceutical ORG on In Vivo Iron Bioavailability

Since ORG expressed higher positive impact on iron bioavailability than RED in the first experiment, ORG was chosen to be further investigated. ORG was given alone to rats with IDA (ORG group), and the results were compared with the control group, which had no treatment and consumed the diet with a low level of iron (Control), as well as with the group consuming a diet with optimum level of iron (Normal) and with the group receiving ORG enriched with iron (ORG + Fe-enriched) (Figure 3).

The idea behind this study was to conclude whether nutraceutical ORG alone or enriched with iron could enhance iron bioavailability and blood iron levels and improve other IDA parameters.

Specifically, body and organ weights of all experimental groups were in accordance with reference values, but the group that was consuming a diet with an optimum level of Fe (Normal group) had the highest body weight compared to other animal groups (Table S4).

At the beginning of the treatment, moderate IDA was confirmed in treated animal groups fed by a low iron diet (Control, ORG, ORG + Fe-enriched) with low Hgb, HCT, RBC, TS, and serum iron (Figure 4, Table S4) [44,45].

Initially, animals across all experimental groups exhibited a rise in WBC counts, which is the same as during the first in vivo study. However, following 28 days of the administered treatments, WBC counts in all experimental animals reverted to reference values. Consequently, serum ferritin was deemed unreliable for further deliberation in the present context [44,45].

The ORG group exhibited the highest increase in TS and serum iron levels, with increments of 1.3 times (29%) and 1.24 times (25%), respectively, compared to the control group. Additionally, the ORG + Fe-enriched group showed even greater increases, with TS and serum iron levels rising by 2.52 times (252%) and 2.64 times (264%), respectively, compared to the control group. Moreover, Hgb and HCT levels experienced a rise of 7.5% and 12%, respectively, in the ORG group, while the ORG + Fe-enriched group demonstrated more substantial increases of 30% and 35%, respectively, compared to the control group (Figure 4, Table S4).

The assessment of the histological features of the duodenum, liver, spleen, and kidney of all groups in this study showed the beneficial effects of ORG (Figures S6–S12).

The normal animal group exhibited the highest iron levels in all examined tissues, followed by tissues from the ORG + Fe-enriched group. Iron levels in tissues from the ORG and control groups were comparable but remained significantly lower than those in the ORG + Fe-enriched group (Figure 4, Table S5).

The gene expression patterns were relatively consistent across the study groups (Control, ORG, and ORG + Fe-enriched). In contrast, the normal group exhibited significant reductions in the expression of most examined mRNAs, except for hepcidin, which showed a marked increase compared to the other groups (Figure 4, Table S6).

## 4. Discussion

The development of the nutritional formula in this study was guided by two principles: (1) ensuring that the final nutraceutical product contains plant foodstuffs naturally rich in iron, and (2) excluding plant foodstuffs containing compounds known to inhibit iron absorption or including them only in trace amounts. Plant polyphenols and phytic acid were identified as compounds that hinder iron absorption. Additionally, the selection of plant foodstuffs was made with careful consideration of their suitability for use in a food processing facility.

Based on the evaluation of polyphenol profiles and phytic acid concentrations, it was determined that all chosen plant-based foodstuffs, as well as the two nutraceuticals (ORG and RED) had sufficient amounts of iron, very low amounts of polyphenols, and adequate amounts of phytic acid. Consequently, it was expected that ORG and RED would not inhibit iron bioavailability.

Specifically, nutraceuticals ORG and RED were made by blending selected plant foodstuffs to ensure that each serving (e.g., 300 mL) contains a minimum of 1 mg of iron, with ORG and RED containing 1.5 and 1 mg per 300 mL, respectively. The achieved iron content per serving was deemed adequate, as iron absorption inhibitors were notably absent in the nutraceuticals. Namely, the recommended daily iron intake ranges between 10 and 20 mg [46]. However, due to the presence of iron absorption inhibitors, only about 10% (up to 2 mg) of this intake is typically absorbed [24]. This aligns closely with the iron content found in the nutraceuticals developed in this study. Also, reputable clinical trials with iron-fortified food (0.63 mg Fe/100 g of tested foodstuffs), containing a similar iron quantity to ORG and RED, have been shown effective in treating IDA [47].

Furthermore, based on the existing literature, it was not possible to predict the exact threshold above which polyphenols present in food will lower iron bioavailability. There is only a general consensus in the scientific literature that high levels of polyphenols significantly reduce iron absorption. Herein, polyphenols that strongly chelate iron, which are flavonoids that have one of the combinations of functional groups, namely 3-hydroxy- and 4-carboxyl group, 5-hydroxy and 4-carboxyl group, or 3′- and 4′-hydroxyl groups (i.e., quercetin, genistein, luteolin, apigenin, epigallocatechin-3-gallate, and their glycosides, etc.), as well as phenolic acids, which have two hydroxyl groups in the ortho position or a hydroxyl and a carboxyl group in the ortho position, were considered negatively. These types of compounds were absent or present in traces in the examined samples. For the sake of comparison, broccoli, a well-known source of iron (9 µg Fe/g raw broccoli) [48], is also a rich source of many polyphenols which are known to chelate iron, such as quercetin and kaempferol (32 µg quercetin and 78 µg kaempferol per g of raw broccoli [36] when summed together present a 30 times greater quantity than that of flavonoids, for example, in KIW (3.8 µg flavonoids/mL KIW)). Hence, broccoli and numerous other fruits and vegetables, acknowledged for their high iron content, are omitted from this study due to their unfavourable profile of iron absorption inhibitors, which could impede iron bioavailability.

Furthermore, while polyphenols are known to inhibit iron absorption, many also possess strong antioxidant properties, which are particularly beneficial during IDA, especially throughout periods of iron supplementation when oxidative stress is elevated. Therefore, it was of particular interest to examine the dual role of polyphenols present in ORG and RED nutraceuticals and, specifically, their potential to support iron absorption by avoiding the formation of non-bioavailable complexes, while simultaneously exerting antioxidant effects against both iron-induced and general ROS. For example, ferulic acid has the capacity to reduce ferric iron (Fe^3+^) without forming stable complexes, thereby maintaining its bioavailability. Chlorogenic acid forms complexes with iron and also reduces it, but these complexes are reversible, allowing iron to be released and absorbed [49]. Furthermore, numerous phenolic compounds modulate transcription factors involved in iron metabolism. For instance, both ferulic acid and quercetin have been shown to activate the Nrf2-signalling pathway, which not only upregulates the expression of antioxidant enzymes but also enhances the transcription of key iron-regulatory proteins, including ferritin heavy (H) and light (L) chains, ferroportin (FPN1), and divalent metal transporter 1 (DMT1). Through this mechanism, these polyphenols can influence cellular iron availability and distribution [21,22,50,51].

The nutraceuticals ORG and RED contained less than 50 mg of phytic acid per serving (48 and 21 mg per serving for ORG and RED, respectively), which is equivalent to 14 mg of phytic acid phosphorus per serving. Given that previous human studies have linked reductions in iron absorption rates with phytic acid amounts exceeding 50 mg per serving [34], it was expected that the concentrations of phytic acid in ORG and RED would not inhibit iron absorption.

Afterward, in order to examine the efficacy of ORG and RED in enhancing iron bioavailability and blood iron levels, as well as improving other main blood IDA parameters when consumed, two sets of in vivo experiments on animals were carried out.

The idea behind the first in vivo study was to conclude whether nutraceuticals ORG and RED could be consumed together with oral iron supplements during IDA treatment and at the same time not to decrease its efficacy.

After treatment, all animal groups, regardless of iron supplementation alone or in combination with ORG and RED, showed improved main blood parameters related to IDA, with similar success seen in Fe sup and ORG + Fe sup groups, while slightly lower success was observed in the RED + Fe sup group.

The amount of iron applied daily in all animal groups within supplementation in this study was 1.24 mg of Fe, from FeSO_4_, which is equivalent to the daily human dose of 60 mg of Fe from FeSO_4_, which is the usual dose for IDA treatment in humans [45,52].

Furthermore, amounts of ORG and RED applied daily in all animal groups were 2.56 mL, which is equivalent to the daily human dose of 120 mL of ORG and RED. This is 2.5 times lesser than the recommended volume for one serving (300 mL).

Within this study, it has been shown that implementing oral treatments on rats having IDA with iron supplementation that is equivalent to the recommended human dose, simultaneously with innovative nutraceuticals ORG and RED, in a daily dose which was 2.5 times lower than the recommended volume for one serving, for 11 consecutive days, led to the same improvement in IDA blood parameters as when oral iron supplementation was applied alone. Thus, nutraceuticals ORG and RED do not lead to a reduction in the positive effect of iron supplementation and could be safe for consumption together with iron supplementation. Since iron supplements are typically recommended to be taken on an empty stomach, the nutraceuticals could be used as a complementary snack to help alleviate gastrointestinal discomfort and potentially improve compliance with iron supplementation therapy. However, this potential benefit should be confirmed in future studies with human volunteers. In addition, one limitation of the study should be acknowledged. The control group (Fe Sup) received a single oral gavage, whereas the ORG Sup and RED Sup groups underwent simultaneous double oral gavage due to the larger volume of treatment required. This difference in administration could have introduced increased stress levels in the experimental groups compared to the control. However, based on nature of the intervention, stress induced by oral gavage is not considered a significant factor influencing iron bioavailability. Therefore, while recognised as a methodological limitation, it is unlikely to have affected the validity of the study outcomes.

The idea behind the second in vivo study was to conclude whether nutraceuticals, when applied alone, would improve iron bioavailability and blood parameters associated with IDA.

The higher mortality rate observed in the first in vivo experiment, compared to the second, was primarily due to the treatment volume being more than twice as high, which led to aspiration pneumonia caused by the inhalation of stomach contents. To prevent this issue in the second experiment, the treatment volume was reduced. Specifically, in the first experiment, the treatment volume was 2.56 mL, while in the second experiment, it was reduced to 1.2 mL.

In the first in vivo experiment, the overall survival rate was 72.2%. The survival rates for each group are as follows: Fe sup—83.3%; ORG + Fe sup—75%; and RED + Fe sup— 58.3%. In contrast, in the second experiment, the average survival rate was 97.3%. The survival rates per group are as follows: Control—100%; Normal—100%; ORG—90%; and ORG + Fe-enriched—100%.

In the second experiment, only the ORG group was selected for further testing. The decision to exclude the RED group was based on the slightly more favourable outcomes observed in the ORG group with respect to the biochemical parameters assessed in the first experiment. The exclusion of the RED group was not related to survival rates or any similar factors.

Furthermore, our aim was to determine whether a longer treatment period with ORG would lead to changes in biochemical parameters in the second experiment. To assess this, a prolonged treatment was conducted over 27 days, compared to the 11-day duration in the initial experiment. In preparation for this extended treatment, we designed a gradual induction protocol to slowly bring the animals into a state of anaemia. This was achieved by progressively reducing the iron content in the diet during the induction phase. As a result, by the end of this phase, the animals were anaemic but maintained a higher body weight, making them better prepared to withstand the 27-day treatment involving everyday gavage. Namely, the average body weight at the start of the 11-day treatment was 184 g, while the average body weight at the beginning of the 28-day treatment was 302 g.

After the treatment, animal groups ORG and ORG + Fe-enriched showed a significant increase in IDA blood parameters, compared with the control group, indicating that treatment was successful, independent of whether ORG was applied alone or enriched with iron. However, the trend of IDA parameters levels was decreasing from the start to the end of the treatment in each group (Control, ORG, and ORG + Fe-enriched). This was expected since treatment lasted 28 days, and during that period, animals were fed with food with a greatly reduced amount of iron. The amount of iron in the applied food was 45 times lower than in regular food, which was applied in the normal group. Thus, ORG alone or enriched with iron could not compensate for such a low amount of dietary iron. Nevertheless, ORG alone or enriched with iron was successful in neutralising the negative effect of low intake of dietary iron and managed to increase IDA blood parameters compared with the control.

Results gained during gene expression analysis in the second experiment are attributed to the presence of IDA in rats [53,54,55,56]. In the liver, hepcidin mRNA exhibited a substantial increase in the normal group, which was approximately 10,000 times higher than all other groups in this study. However, this should be interpreted as a significant decrease in the control, ORG, and ORG + Fe-enriched groups, considering that hepcidin levels are considered regular in the normal group. This phenomenon aligns with the occurrence of IDA in these three groups, wherein during IDA, hypoxia and increased demand for erythropoiesis lead to a reduction in hepcidin expression to facilitate efficient iron mobilisation. An increase in hepcidin mRNA expression was observed in the normal group in the kidney and spleen tissues, although the difference was less pronounced.

In general, the efficacy of treatment was notably higher in the ORG + Fe-enriched group compared to the ORG group, as anticipated due to the additional iron administered in the former. The daily iron dose in the ORG + Fe-enriched group amounted to 0.11 mg of Fe from the FeSO_4_ source, which is equivalent to a daily human dose of 0.29 mg of Fe from the FeSO_4_ source. This dosage is 200 times lower than the recommended dose for the treatment of IDA in humans [45,52]. Nevertheless, it was proven to be sufficient for the effective treatment of IDA. This finding serves as additional confirmation that the ORG does not impact the bioavailability of iron when consumed simultaneously with iron supplementation.

The daily administration of ORG alone or enriched with iron in this in vivo study amounted to 1.2 mL of concentrated ORG. This dose is equivalent to 1.56 mL of the non-concentrated, standard ORG serving applied in the previous experiment described herein. Translated to a human dose, this dose corresponds to a daily human dose of 41 mL of ORG, which is 7.3 times lesser than the quantity found in a single serving (300 mL).

Regarding iron supplementation, the nutritional formula of ORG + Fe-enriched provided an additional daily iron intake of 0.11 mg of Fe from FeSO_4_. Translated to a human dose, this is equivalent to a daily dose of 2.72 mg of Fe from FeSO_4_, which is 22 times lesser than the established dose for the treatment of IDA in humans, set at 60 mg of Fe from FeSO_4_ [42,45]. Thus, ORG + Fe-enriched could be considered as an iron-fortified drink [57].

## 5. Conclusions

In this study, it has been demonstrated that the oral administration of the innovative nutraceutical ORG, both in its standalone form or enriched with iron, over a period of 28 consecutive days, yielded significant improvements in blood parameters associated with IDA in rats. It was anticipated that the extended duration of the treatment would result in further enhancement in blood parameters. Consequently, this study highlights that the innovative plant-based nutraceutical contains highly bioavailable iron and has substantial potential as a viable alternative for the enhancement of blood iron level and improvement in biochemical parameters associated with IDA. Thus, the innovative nutraceutical could be useful in treatment of mild to moderate IDA in humans or for protective measures during periods when the likelihood of IDA occurrence is heightened, such as during reproductive phases in women, pregnancy, and growth periods in children. Further studies are needed to confirm these findings in human models and to investigate the role of other naturally occurring enhancers of iron absorption, such as ascorbic, citric, malic, and tartaric acids, including their ratios to iron and to iron absorption inhibitors, such as phytic acid and polyphenols.

Overall, this study affirms the possibility of developing a nutritional formula that serves as a versatile foundation for creating various nutraceuticals, including juices, smoothies, bars, porridges, ice creams, soups, etc., for the prevention and treatment of IDA. This strategy is in accordance with the contemporary initiatives set forth by the WHO and UNICEF for the effective management of IDA, aiming to uphold the health and well-being of children, adolescents, and women [2].

## 6. Patents

The details of the chemical composition of innovative plant-based nutraceuticals for increasing blood iron levels, e.g., prevention and treatment of anaemia in humans, especially iron deficiency anaemia (IDA), are a subject of the patent application no. PCT/EP2024/051077.

## Figures and Tables

**Table 2:** Total phenolic, flavonoid, phytic acid and iron contents ^a^ in selected plant-based foodstuffs and nutraceuticals (ORG and RED).

	Plant-Based Foodstuffs								Nutraceuticals	
	WPB	BEE	KIW	PIN	BSB	MEL	CIN	HON	ORG	RED
Total phenolics (µg gallic acid equivalents/g of plant foods or mL of juice/smoothie)	259.09 ± 13.98	683.45 ± 34.65	315.07 ± 2.16	538.60 ± 34.53	112.87 ± 9.70	180.66 ± 13.04	(36.56 ± 2.26) × 10^3^	191.22 ± 2.61	360.06 ± 5.04 b ^b^	988.42 ± 43.65 a
Total flavonoids (µg quercetin equivalents/g of plant foods or mL of juice/smoothie)	68.26 ± 3.66	109.33 ± 7.28	6.17 ± 0.57	<4.28	102.20 ± 4.99	243.17 ± 10.56	7184.82 ± 655.33	<30.61	4.63 ± 0.10 b	6.16 ± 0.67 a
Total phytic acid (mg phytic acid/g of plant foods or mL of juice/smoothie)	0.43 ± 0.03	<DL ^c^	0.07 ± 0.00	0.60 ± 0.05	0.58 ± 0.03	0.76 ± 0.05	2.77 ± 0.18	2.35 ± 0.22	0.16 ± 0.01 a	0.07 ± 0.00 b
Total iron (µg iron/g of plant foods or mL of juice/smoothie)	5.66 ± 0.52	2.98 ± 0.26	4.20 ± 0.26	4.31 ± 0.26	2.47 ± 0.24	1.73 ± 0.17	259.11 ± 23.25	2.41 ± 0.16	5.24 ± 0.47 a	3.51 ± 0.16 b
DPPH assay (mM Trolox equivalents/g of plant foods or mL of juice/smoothie)	0.23 ± 0.00	1.07 ± 0.16	0.18 ± 0.01	0.67 ± 0.01	0.15 ± 0.00	0.35 ± 0.03	42.84 ± 2.64	N/A	0.37 ± 0.04 b	0.50 ± 0.02 a
FRAP assay (mM Trolox equivalents/g of plant foods or mL of juice/smoothie)	0.18 ± 0.01	3.53 ± 0.11	0.44 ± 0.03	0.78 ± 0.03	0.10 ± 0.01	0.61 ± 0.02	160.59 ± 2.65	0.12 ± 0.01	0.31 ± 0.02 b	0.41 ± 0.02 a

^a^ Values are means ± SEM of three measurements. ^b^ Means within each row with different letters (a–b) differ significantly (*p* ≤ 0.05). ^c^ Below detection limit (DL), which is 0.40 mg phytic acid/g. Abbreviations: BEE: beetroot cold-pressed juice; BSB: butternut squash boiled; CIN: cinnamon powder; HON: acacia honey; KIW: kiwi cold-pressed juice; MEL: melon cold-pressed juice; ORG: nutraceutical, named orange smoothie; PIN: pineapple cold-pressed juice; RED: nutraceutical, named red smoothie; WPB: white potato boiled; N/A: not active.

## Data Availability

This study was carried out using publicly available data from SCOPUS (https://www.scopus.com, accessed on 27 October 2025), PubMed (https://pubmed.ncbi.nlm.nih.gov), USDA Database for the Flavonoid Content of Selected Foods. Release 3.3 (https://doi.org/10.15482/USDA.ADC/1529181), Phenol-Explorer (http://phenol-explorer.eu, accessed on 27 October 2025), USDA National Nutrient Database for Standard Reference Legacy (https://doi.org/10.15482/USDA.ADC/1529216), https://ods.od.nih.gov/factsheets/Iron-Consumer/ and https://fdc.nal.usda.gov/fdc-app.html#/?component=1089 (accessed on 27 October 2025) and https://www.taconic.com/rat-model/wistar-hannover-galas (accessed on 27 October 2025). The original contributions presented in this study are included in the article/supplementary material. Further inquiries can be directed to the corresponding author.

## Supplementary Materials

The following supporting information can be downloaded at https://www.mdpi.com/article/10.3390/antiox14111335/s1, Figure S1: The HPLC-DAD chromatograms of polyphenolic compounds from selected plant-based foodstuffs at 340 nm; Figure S2: Photomicrographs of duodenal tissue of rats with IDA after simultaneous treatment with nutraceuticals (ORG and RED) and iron supplementation (H&E, 5×); Figure S3: Photomicrographs of liver tissue of rats with IDA after simultaneous treatment with nutraceuticals (ORG and RED) and iron supplementation (H&E, 10× (A–C), 20× (D–F)); Figure S4: Photomicrographs of spleen section of rats with IDA after simultaneous treatment with nutraceuticals (ORG and RED) and iron supplementation (H&E, 10× (A–C), 20× (D–F)); Figure S5: Photomicrographs of kidney section of rats with IDA after simultaneous treatment with nutraceuticals (ORG and RED) and iron supplementation. (H&E, 10×); Figure S6: Photomicrographs of duodenal tissue of rats with IDA after treatment with nutraceutical ORG alone and enriched with iron (H&E, 5×); Figure S7: Photomicrographs of liver tissue of rats; Figure S8: Photomicrographs of liver tissue of rats; Figure S9: Photomicrographs of liver tissue of rats; Figure S10: Photomicrographs of liver tissue of rats; Figure S11: Photomicrographs of spleen tissue of rats; Figure S12: Photomicrographs of kidney tissue of rats with IDA after treatment with nutraceutical ORG alone and enriched with iron (H&E, 20×); Table S1: Body weight, organ weight, and selected IDA blood parameters of rats before and after the simultaneous treatment with nutraceuticals (ORG and RED) and iron supplementation; Table S2: Total non-haem iron content in tissues of rats with IDA simultaneously treated with nutraceuticals (ORG and RED) and iron supplementation; Table S3: Effect of simultaneous treatment with nutraceuticals (ORG and RED) and iron supplementation on the expression of chosen mRNA of rats with IDA; Table S4: Body weight, organ weight, and selected IDA blood parameters of rats before and after treatment with nutraceutical ORG alone and enriched with iron; Table S5: Total non-haem iron in tissues of rats with IDA treated with nutraceutical ORG alone and enriched with iron; Table S6: Effect of nutraceutical ORG alone and enriched with iron on expression of chosen mRNA of rats with IDA; Table S7: PCR primers used in the study. References [58,59,60,61,62,63,64] are cited in Supplementary Materials.

## Abbreviations

The following abbreviations are used in this manuscript:

BEE	beetroot cold-pressed juice
BSB	butternut squash boiled
CIN	cinnamon powder
Dcytb	duodenal cytochrome B
DL	below detection limit
DMT1	divalent metal transporter 1
FPN	ferroportin
GAPDH	glyceraldehyde 3-phosphate dehydrogenase
H&E	hematoxylin and eosin
HCT	haematocrit
Hgb	haemoglobin
HON	acacia honey
HPF	high power fields
IDA	iron deficiency anaemia
IP	intraperitoneal injection
KIW	kiwi cold-pressed juice
LoQ	below instrument quantification limit
MCH	mean corpuscular haemoglobin
MCHC	mean corpuscular haemoglobin concentration
MCV	mean corpuscular volume
MEL	melon cold-pressed juice
ORG	final food product, named orange smoothie
PIN	pineapple cold-pressed juice
PLT	platelet count
RBC	red blood cells
RDW	red cell distribution width
RED	final food product, named red smoothie
SEM	standard error of the mean
TIBC	total iron-binding capacity
TS	transferrin saturation
UIBC	unsaturated iron-binding capacity
WBC	white blood cell
WHO	World Health Organisation
WPB	white potato boiled
