# Supplementary material for: Innovative Plant-Based Nutraceuticals: Enhancing Iron Bioavailability to Address Iron Deficiency Anaemia
Source: Antioxidants (Basel). 2025 Nov 5;14(11):1335. doi: 10.3390/antiox14111335 (PMC12649512; doi:10.3390/antiox14111335)
Supplement: Supplementary file 1 [file antioxidants-14-01335-s001.zip › antioxidants-3914324-supplementary.pdf]

antioxidants

Article

Innovative Plant-Based Nutraceuticals: Enhancing Iron Bioavailability to Address Iron Deficiency Anaemia

Nemanja Živanović [1], Vesna Mijatović Jovin [2], Bojana Andrejić Višnjić [2], Diandra Pintać Šarac [2], Danica Ćujić [3], Nataša Simin [1] and Marija Lesjak [1,*]

[1] Faculty of Sciences University of Novi Sad, Department of Chemistry, Biochemistry and Environmental Protection, Trg Dositeja Obradovica 3, 21000 Novi Sad, Serbia
[2] Faculty of Medicine University of Novi Sad, Hajduk Veljkova 3, 21137 Novi Sad, Serbia
[3] Institute for the Application of Nuclear Energy, University of Belgrade, Banatska 31b, 11080 Zemun, Serbia
* Correspondence: e-mail: marija.lesjak@dh.uns.ac.rs; phone: +381214852755; fax: +38121454065

SUPPLEMENTARY MATERIAL

 https://doi.org/10.3390/antiox14111335

Table S1. Body weight, organ weight and selected IDA blood parameters of rats before and after the simultaneous treatment with nutraceuticals (ORG and RED) and iron supplementation

Parameter		Animal group: **Fe sup** (12 animals at the start 10 animals at the end of experiment)	**ORG + Fe sup** (12 animals at the start 9 animals at the end of experiment)	**RED + Fe sup** (12 animals at the start 7 animals at the end of experiment)	Physiological values and reference
Body weight (g)	At the start of treatment (7 weeks old)	192 ± 17[a] a	196 ± 17 a	164 ± 16 b	173–252 g [58]
	At the end of treatment (9 weeks old)	233 ± 23 a	243 ± 20 a	232 ± 21 a	234–334 g [58]
Organ weight at the end of experiment (g)	heart	0.81 ± 0.06 b	0.90 ± 0.09 a	0.93 ± 0.07 a	≈ 0.30% body weight [59]
	liver	9.48 ± 0.90 b	10.63 ± 0.89 a	9.45 ± 0.81 b	≈ 2.84% body weight [59]
	spleen	0.53 ± 0.11 b	0.52 ± 0.08 b	0.72 ± 0.08 a	≈ 0.23% body weight [59]
	kidney	0.87 ± 0.09 b	1.00 ± 0.11 a	1.05 ± 0.10 a	≈ 0.36% body weight [59]
WBC ($\times 10^3/\mu L$)	At the start of the treatment	14.46 ± 1.89 a	12.59 ± 1.22 b	15.10 ± 1.87 a	1.96–8.25 × $10^3/\mu L$ [60]
Lymphocytes ($\times 10^3/\mu L$)		11.39 ± 1.18 a	10.00 ± 0.90 b	11.96 ± 1.11 a	1.41–7.11 × $10^3/\mu L$ [60]
Monocytes ($\times 10^3/\mu L$)		0.34 ± 0.12 a	0.21 ± 0.03 b	0.36 ± 0.07 a	0.03–0.18 × $10^3/\mu L$ [60]
Granulocytes ($\times 10^3/\mu L$)		3.09 ± 0.59 a	2.05 ± 0.51 b	2.63 ± 0.57 a	0.23–1.78 × $10^3/\mu L$ [60]

 https://doi.org/10.3390/antiox14111335

Lymphocytes (%)		75.76 ± 3.60 b	78.95 ± 2.86 a	78.08 ± 3.92 a	66.6–90.3 % [60]
Monocytes (%)		2.54 ± 0.24 a	2.33 ± 0.39 a	2.43 ± 0.51 a	0.8–3.8 % [60]
Granulocytes (%)		21.96 ± 2.03 a	19.40 ± 1.66 b	19.97 ± 2.61 ab	7.2–31 % [60]
PLT (×10^3/μL)		837.43 ± 58.67 b	939.43 ± 145.31 ab	982.63 ± 122.10 a	638–1177 ×10^3/μL [60]
Hgb (g/dL)		10.14 ± 0.94 a	9.99 ± 0.80 a	10.08 ± 0.84 a	13.7–17.6 g/dL [60]
HCT (%)		28.68 ± 2.88 a	27.59 ± 2.14 a	28.77 ± 2.79 a	39.6–52.5 % [60]
RBC (× 10^6/μL)		5.79 ± 0.49 a	5.63 ± 0.38 a	5.80 ± 0.53 a	7.27–9.68 × 10^6/μL [60]
MCV (fL)		48.29 ± 4.50 a	49.09 ± 2.08 a	47.63 ± 4.79 a	48.9–57.9 fL [60]
MCH (pg)		17.34 ± 1.56 a	17.69 ± 0.75 a	16.84 ± 1.57 a	17.1–20.4 pg [60]
MCHC (g/dL)		35.99 ± 0.47 a	36.15 ± 0.31 a	36.32 ± 1.79 a	32.9–37.5 g/dL [60]
RDW (%)		32.33 ± 3.51 a	27.03 ± 2.29 b	33.82 ± 3.69 a	11.1–15.2 % [58]
Serum iron (μmol/L)		5.66 ± 0.60 b	6.37 ± 0.76 a	5.04 ± 0.66 b	25–36 μmol/L [61-63]
UIBC (μmol/L)		151.34 ± 2.33 a	151.21 ± 1.16 a	150.88 ± 3.57 a	25–36 μmol/L [61-63]
TIBC (μmol/L)		157.64 ± 2.78 a	157.51 ± 2.48 a	156.82 ± 3.75 a	50–72 μmol/L [61-63]
TS (%)		3.61 ± 0.38 ab	4.03 ± 0.46 a	3.24 ± 0.46 b	30–40% [62-63]
Serum ferritin		/[b]	/	/	≈ 423 ng/mL [64]
Serum transferrin (mg/L)		716.07 ± 28.25 b	742.35 ± 16.54 a	740.18 ± 25.93 a	≈ 1600 mg/L [64]
WBC (×10^3/μL)	At the end of the treatment	1.7 ± 0.14 b	1.12 ± 0.34 c	2.33 ± 1.14 a	1.96–8.25 × 10^3/μL [60]
Lymphocytes (×10^3/μL)		0.96 ± 0.26 a	0.66 ± 0.21 b	1.02 ± 0.34 ab	1.41–7.11 × 10^3/μL [60]
Monocytes (×10^3/μL)		0.10 ± 0.00 a	0.10 ± 0.00 a	0.22 ± 0.15 a	0.03–0.18 × 10^3/μL [60]
Granulocytes (×10^3/μL)		0.54 ± 0.25 ab	0.48 ± 0.23 b	1.06 ± 0.61 a	0.23–1.78 × 10^3/μL [60]
Lymphocytes (%)		67.34 ± 6.47 a	68.93 ± 5.26 a	61.93 ± 7.93 a	66.6–90.3 % [60]
Monocytes (%)		2.79 ± 0.30 a	3.11 ± 0.53 a	3.08 ± 0.60 a	0.8–3.8 % [60]
Granulocytes (%)		29.26 ± 4.37 a	29.20 ± 4.12 a	43.17 ± 9.93 a	7.2–31 % [60]
PLT (×10^3/μL)		1592.20 ± 366.13 ab	1284 ± 86.05 a	1844.86 ± 258.53 b	638–1177 ×10^3/μL [60]
Hgb (g/dL)		14.61 ± 0.98 a	13.38 ± 0.85 b	12.62 ± 0.92 b	13.7–17.6 g/dL [60]
HCT (%)		42.14 ± 2.74 a	38.77 ± 2.40 b	36.95 ± 2.73 b	39.6–52.5 % [60]
RBC (× 10^6/μL)		7.47 ± 0.52 a	6.98 ± 0.40 b	7.01 ± 0.49 ab	7.27–9.68 × 10^6/μL [60]
MCV (fL)		56.55 ± 2.79 a	55.61 ± 1.78 a	56.89 ± 4.49 a	48.9–57.9 fL [60]
MCH (pg)		19.51 ± 0.86 a	19.11 ± 0.62 a	19.03 ± 1.65 a	17.1–20.4 pg [60]

MCHC (g/dL)		34.60 ± 0.57 a	34.47 ± 0.34 a	34.13 ± 0.35 a	32.9–37.5 g/dL [60]
RDW (%)		18.17 ± 1.99 a	19.98 ± 3.76 a	18.86 ± 2.08 a	11.1–15.2 % [58]
Serum iron (μmol/L)		30.40 ± 4.94 a	29.82 ± 5.26 a	20.68 ± 1.73 b	25–36 μmol/L [61-63]
UIBC (μmol/L)		74.16 ± 14.29 b	67.10 ± 10.06 b	82.40 ± 8.17 a	25–36 μmol/L [61-63]
TIBC (μmol/L)		101.02 ± 11.33 ab	96.38 ± 9.78 b	110.57 ± 10.61 a	50–72 μmol/L [61-63]
TS (%)		38.17 ± 13.00 a	29.35 ± 6.21 a	20.99 ± 3.20 b	30–40% [62-63]
Serum ferritin (ng/mL)		199.93 ± 80.63 a	96.71 ± 36.86 b	304.88 ± 163.47 a	≈ 423 ng/mL [64]
Serum transferrin (mg/L)		748.09 ± 20.54 b	770.74 ± 15.17 a	749.03 ± 30.06 b	≈ 1600 mg/L [64]

[a] Values are means ± SEM. Means within each row with different letters (a-c) differ significantly (p ≤ 0.05).

[b] Measurement was not performed due to low amount of serum sample.

Abbreviations: HCT: haematocrit, Hgb: haemoglobin, MCH: mean corpuscular haemoglobin, MCHC: mean corpuscular haemoglobin concentration, MCV: mean corpuscular volume, PLT: platelet count, RBC: red blood cells, RDW: red cell distribution width, TIBC: total iron binding capacity, TS: transferrin saturation, UIBC: unsaturated iron-binding capacity, WBC: white blood cell.

Table S2. Total non-haem iron[a] content in tissues of rats with IDA simultaneously treated with nutraceuticals (ORG and RED) and iron supplementation

Animal group	liver	spleen	duodenum
	μg non-haem iron/g dry tissue		
**Fe sup** (10 animals at the end of experiment)	564.60 ± 44.56 b[b]	316.93 ± 30.49 a	27.93 ± 2.65 b
**ORG + Fe sup** (9 animals at the end of experiment)	722.50 ± 71.08 a	321.95 ± 32.41 a	34.62 ± 3.46 a
**RED + Fe sup** (7 animals at the end of experiment)	598.84 ± 57.72 b	258.20 ± 23.96 b	34.10 ± 3.28 a

[a] Values are means ± SEM.

[b] Means within each column with different letters (a-b), differ significantly (p ≤ 0.05).

Table S3. Effect of simultaneous treatment with nutraceuticals (ORG and RED) and iron supplementation on the expression of chosen mRNA of rats with IDA

Animal group			**Fe sup** (10 animals at the end of experiment)	**ORG + Fe sup** (9 animals at the end of experiment)	**RED + Fe sup** (7 animals at the end of experiment)
Fold expression change of mRNA compared to GAPDH mRNA	Duodenum	DMT1	0.98 ± 0.88 c[a]	2.21 ± 0.63 b	6.47 ± 0.99 a
		Dcytb	0.84 ± 0.46 b	1.06 ± 0.63 ab	1.88 ± 0.87 a
		FPN	0.95 ± 0.30 b	1.46 ± 0.27 a	0.93 ± 0.53 ab
		Hephaestin	1.13 ± 0.31 a	1.05 ± 0.18 a	1.15 ± 0.34 a
		Ferritin	1.02 ± 0.18 b	1.30 ± 0.35 a	0.75 ± 0.21 c
	Liver	Hepcidin	0.78 ± 0.90 b	8.54 ± 2.90 a	0.58 ± 0.60 b
		FPN	0.97 ± 0.28 a	1.11 ± 0.27 a	0.97 ± 0.18 a
		Ferritin	0.91 ± 0.35 a	1.11 ± 0.38 a	1.11 ± 0.36 a
	Spleen	Hepcidin	0.93 ± 0.19 a	0.86 ± 0.29 a	0.79 ± 0.16 a
		FPN	0.81 ± 0.24 a	0.52 ± 0.18 b	0.53 ± 0.09 b
		Ferritin	1.02 ± 0.22 a	1.14 ± 0.25 a	0.70 ± 0.15 b
	Kidney	Hepcidin	1.09 ± 0.62 a	0.93 ± 0.26 a	0.57 ± 0.14 b

[a] Values are means ± SEM. Means within each row with different letters (a-c), differ significantly ($p \leq 0.05$).

Abbreviation: Dcytb: duodenal cytochrome B; DMT1: divalent metal transporter 1; FPN: ferroportin; GAPDH: glyceraldehyde 3-phosphate dehydrogenase.

Table S4. Body weight, organ weight and selected IDA blood parameters of rats before and after treatment with nutraceutical ORG alone and enriched with iron

Parameter		Animal group				Physiological values and reference
		**⚜ontrol** (11 animals at the start 11 animals at the end of experiment)	**Normal** (6 animals at the start 6 animals at the end of experiment)	**ORG** (10 animals at the start 9 animals at the end of experiment)	**ORG + Fe enrich** (10 animals at the start 10 animals at the end of experiment)	
Body weight (g)	At the start of treatment (10 weeks old)	286.00 ± 26.68[a] d	366.83 ± 31.11 a	300.00 ± 28.91 cd	323.30 ± 31.40 bc	173–252 g [58]
	At the end of treatment (14 weeks old)	378.20 ± 37.58 b	479.33 ± 37.18 a	368.00 ± 28.36 b	398.30 ± 27.81 b	234–334 g [58]
Organ weight at the end of experiment (g)	heart	1.12 ± 0.11 ab	1.42 ± 0.27 a	1.09 ± 0.10 b	1.24 ± 0.14 a	≈ 0.30% body weight [59]
	liver	11.74 ± 1.07 b	15.03 ± 1.33 a	11.62 ± 1.65 b	14.264 ± 1.41 a	≈ 2.84% body weight [59]
	spleen	0.99 ± 0.09 c	1.38 ± 0.07 a	1.02 ± 0.08 c	1.18 ± 0.10 b	≈ 0.23% body weight [59]
	kidney	0.61 ± 0.13 a	0.73 ± 0.09 a	0.67 ± 0.15 a	0.68 ± 0.11 a	≈ 0.36% body weight [59]
WBC (×10^3/μL)	At the start of the treatment	10.70 ± 1.26 a	9.36 ± 1.75 a	10.85 ± 1.12 a	10.26 ± 1.30 a	1.96–8.25 × 10^3/μL [60]
Lymphocytes (×10^3/μL)		7.44 ± 0.70 ab	6.36 ± 1.70 b	7.83 ± 0.92 a	6.90 ± 1.21 ab	1.41–7.11 × 10^3/μL [60]
Monocytes (×10^3/μL)		0.36 ± 0.05 a	0.46 ± 0.13 a	0.37 ± 0.10 a	0.43 ± 0.10 a	0.03–0.18 × 10^3/μL [50]
Granulocytes (×10^3/μL)		2.35 ± 0.28 b	2.68 ± 0.44 a	2.89 ± 0.59 ab	2.42 ± 0.69 ab	0.23–1.78 × 10^3/μL [60]
Lymphocytes (%)		72.45 ± 4.26 a	68.33 ± 4.50 a	71.17 ± 4.39 a	68.76 ± 5.75 a	66.6–90.3 % [60]
Monocytes (%)		3.51 ± 0.60 c	4.42 ± 0.56 ab	3.79 ± 0.63 bc	4.54 ± 0.85 a	0.8–3.8 % [60]
Granulocytes (%)		23.64 ± 2.25 a	26.82 ± 3.82 a	26.44 ± 3.10 a	26.53 ± 3.77 a	7.2–31 % [60]
PLT (×10^3/μL)		829.44 ± 128.77 a	586.20 ± 73.14 bc	718.71 ± 194.33 ac	726.38 ± 207.07 ac	638–1177 ×10^3/μL [60]
Hgb (g/dL)		12.12 ± 1.03 a	12.06 ± 1.12 a	11.41 ± 0.57 a	12.54 ± 1.91 a	13.7–17.6 g/dL [60]
HCT (%)		35.55 ± 2.99 a	36.42 ± 3.57 a	33.83 ± 1.91 a	32.49 ± 3.99 a	39.6–52.5 % [60]
RBC (× 10^6/μL)		6.78 ± 0.58 a	6.33 ± 0.41 ac	6.45 ± 0.43 ac	6.07 ± 0.70 bc	7.27–9.68 × 10^6/μL [60]
MCV (fL)		52.20 ± 2.61 b	59.65 ± 1.20 a	52.61 ± 2.15 b	53.52 ± 1.86 b	48.9–57.9 fL [60]

MCH (pg)		17.85 ± 0.81 b	19.72 ± 0.57 a	17.68 ± 0.85 b	17.81 ± 0.63 b	17.1–20.4 pg [60]
MCHC (g/dL)		34.31 ± 0.69 a	33.12 ± 0.44 c	33.71 ± 0.61 b	33.36 ± 0.46 bc	32.9–37.5 g/dL [60]
RDW (%)		18.63 ± 2.07 a	14.83 ± 0.82 b	18.02 ± 1.84 a	18.18 ± 2.11 a	11.1–15.2 % [61]
Serum iron (μmol/L)		9.93 ± 1.96 b	35.00 ± 2.24 a	10.02 ± 1.87 b	9.70 ± 1.92 b	25–36 μmol/L [61-63]
UIBC (μmol/L)		99.54 ± 9.76 a	77.27 ± 5.39 b	96.63 ± 8.22 a	97.94 ± 9.62 a	25–36 μmol/L [61-63]
TIBC (μmol/L)		109.28 ± 89.05 a	111.00 ± 6.22 a	108.14 ± 6.11 a	110.05 ± 7.80 a	50–72 μmol/L [61-63]
TS (%)		8.81 ± 1.58 b	30.39 ± 2.95 a	8.80 ± 0.69 b	8.75 ± 2.01 b	30–40% [62-63]
Serum ferritin (ng/mL)		389.38 ± 50.61 a	295.10 ± 62.36 b	442.82 ± 82.06 a	426.85 ± 80.54 a	≈ 423 ng/mL [64]
Serum transferrin (mg/L)		762.27 ± 14.26 b	782.95 ± 14.10 a	748.49 ± 24.95 a	751.48 ± 16.96 a	≈ 1600 mg/L [64]
WBC (×10³/μL)	At the end of the treatment	2.1 ± 0.30 a	4.10 ± 1.13 a	3.05 ± 0.90 b	2.20± 0.80 a	1.96–8.25 × 10³/μL [60]
Lymphocytes (×10³/μL)		1.59 ± 0.48 ab	3.10 ± 0.79 a	2.08 ± 0.72 a	1.48 ± 0.45 b	1.41–7.11 × 10³/μL [60]
Monocytes (×10³/μL)		0.10 ± 0.00 a	0.10 ± 0.00 a	0.10 ± 0.00 a	0.10 ± 0.00 a	0.03–0.18 × 10³/μL [60]
Granulocytes (×10³/μL)		0.70 ± 0.09 a	0.95 ± 0.25 a	0.59 ± 0.32 a	0.64 ± 0.26 a	0.23–1.78 × 10³/μL [60]
Lymphocytes (%)		62.29 ± 4.78 b	73.50 ± 3.51 a	66.44 ± 6.34 b	67.68 ± 6.45 b	66.6–90.3 % [60]
Monocytes (%)		2.63 ± 0.22 b	2.55 ± 0.30 b	2.70 ± 0.30 b	3.34 ± 0.73 a	0.8–3.8 % [60]
Granulocytes (%)		34.42 ± 4.60 a	24.20 ± 3.20 bc	30.57± 4.76 ab	28.10 ± 4.67 bc	7.2–31 % [60]
PLT (×10³/μL)		1171.78 ± 110.02 a	770.25 ± 48.53 c	1050.88 ± 109.97 b	1201.88 ± 133.30 a	638–1177 ×10³/μL [60]
Hgb (g/dL)		8.92 ± 0.43 d	13.93 ± 0.60 a	9.60 ± 0.79 c	11.66 ± 1.00 b	13.7–17.6 g/dL [60]
HCT (%)		24.89 ± 1.11 d	41.65 ± 3.07 a	27.91 ± 2.52 c	33.66 ± 3.73 b	39.6–52.5 % [60]
RBC (× 10⁶/μL)		6.38 ± 0.38 b	7.30 ± 0.38 a	6.74 ± 0.47 b	7.45 ± 0.57 a	7.27–9.68 × 10⁶/μL [60]
MCV (fL)		39.19 ± 2.13 d	56.37 ± 0.82 a	42.56 ± 3.72 c	45.94 ± 3.23 b	48.9–57.9 fL [60]
MCH (pg)		13.94 ± 0.78 c	19.05 ± 0.24 a	14.21 ± 0.70 c	15.46 ± 1.08 b	17.1–20.4 pg [60]
MCHC (g/dL)		35.79 ± 0.81 a	34.027 ± 0.40 bc	34.56 ± 0.45 b	33.83 ± 0.62 c	32.9–37.5 g/dL [60]
RDW (%)		25.10 ± 2.03 a	13.65 ± 0.52 c	23.74 ± 1.56 a	20.68 ± 2.27 b	11.1–15.2 % [58]
Serum iron (μmol/L)		5.80 ± 0.89 d	29.02 ± 2.14 a	7.22 ± 0.64 c	15.34 ± 2.67 b	25–36 μmol/L [61-63]
UIBC (μmol/L)		118.47 ± 7.29 a	72.78 ± 4.56 c	111.70 ± 8.56 a	96.26 ± 10.96 b	25–36 μmol/L [61-63]
TIBC (μmol/L)		124.30 ± 7.25 a	98.78 ± 7.70 c	117.70 ± 9.47 ab	110.47 ± 8.47 b	50–72 μmol/L [61-63]
TS (%)		4.83 ± 0.71 d	28.13 ± 1.57 a	6.32 ± 1.09 c	12.34 ± 2.74 b	30–40% [62-63]
Serum ferritin (ng/mL)		558.92 ± 50.70 a	363.73 ± 46.68 b	554.42 ± 52.43 a	508.67 ± 64.94 a	≈ 423 ng/mL [64]

Serum transferrin (mg/L)		727.12 ± 58.14 b	776.98 ± 20.59 a	750.59 ± 28.02 b	777.93 ± 16.05 a	≈ 1600 mg/L [64]

[a] Values are means ± SEM. Means within each row with different letters (a-d) differ significantly ($p \leq 0.05$).

Abbreviations: HCT: haematocrit, Hgb: haemoglobin, MCH: mean corpuscular haemoglobin, MCHC: mean corpuscular haemoglobin concentration, MCV: mean corpuscular volume, PLT: platelet count, RBC: red blood cells, RDW: red cell distribution width, TIBC: total iron binding capacity, TS: transferrin saturation, UIBC: unsaturated iron-binding capacity, WBC: white blood cell.

Table S5. Total non-haem iron[a] in tissues of rats with IDA treated with nutraceutical ORG alone and enriched with iron

Animal group	liver	spleen	duodenum
	µg non-haem iron/g dry tissue		
**Control** (11 animals at the end of experiment)	121.67 ± 11.60 d[b]	296.89 ± 24.35 c	24.46 ± 2.05 b
**Normal** (6 animals at the end of experiment	404.69 ± 17.95 a	1253.24 ±91.73 a	102.23 ± 9.91 a
**ORG** (9 animals at the end of experiment	141.59 ± 13.87 c	298.25 ± 28.38 c	24.90 ± 2.28 b
**ORG + Fe enrich** (10 animals at the end of experiment	173.17 ± 15.24 b	367.74 ± 34.70 b	26.35 ± 2.51 b

[a] Values are means ± SEM.

[b] Means within each column with different letters (a-d), differ significantly ($p \leq 0.05$).

Table S6. Effect of nutraceutical ORG alone and enriched with iron on expression of chosen mRNA of rats with IDA

			Control (11 animals at the end of experiment)	Normal (6 animals at the end of experiment)	ORG (9 animals at the end of experiment)	ORG + Fe enrich (10 animals at the end of experiment)
Fold expression change of mRNA compared to GAPDH mRNA	Duodenum	DMT1	1.17 ± 0.74[a] a	0.01 ± 0.01 b	1.73 ± 0.58 a	1.17 ± 0.82 a
		Dcytb	1.20 ± 0.67 a	0.01 ± 0.01 c	0.96 ± 0.46 ab	0.61 ± 0.51 b
		FPN	1.32 ± 0.48 b	0.23 ± 0.15 c	1.83 ± 0.39 a	1.24 ± 0.41 b
		Hephaestin	0.61 ± 0.28 a	0.11 ± 0.05 c	0.65 ± 0.12 a	0.37 ± 0.24 b
		Ferritin	0.99 ± 0.30 a	0.22 ± 0.09 c	0.93 ± 0.14 a	0.42 ± 0.22 b
	Liver	Hepcidin	1.18 ± 0.71 c	10749.91 ± 1080.92 a	2.05 ± 0.59 b	2.25 ± 1.00 b
		FPN	1.66 ± 0.60 a	0.56 ± 0.30 b	1.23 ± 0.50 a	0.52 ± 0.28 b
		Ferritin	2.61 ± 0.65 a	0.31 ± 0.12 b	2.24 ± 0.75 a	0.44 ± 0.28 b
	Spleen	Hepcidin	1.53 ± 0.40 b	3.39 ± 1.12 a	1.66 ± 0.24 b	1.42 ± 0.53 b
		FPN	1.41 ± 0.31 a	0.60 ± 0.27 b	1.48 ± 0.38 a	1.30 ± 0.42 a
		Ferritin	1.70 ± 0.50 a	0.53 ± 0.19 c	1.29 ± 0.29 b	1.32 ± 0.31 b
	Kidney	Hepcidin	0.96 ± 0.30 a	1.80 ± 0.80 a	0.88 ± 0.62 a	1.12 ± 0.93 a

[a] Values are means ± SEM. Means within each row with different letters (a-c), differ significantly ($p \le 0.05$).

Abbreviation: Dcytb: duodenal cytochrome B; DMT1: divalent metal transporter 1; FPN: ferroportin; GAPDH: glyceraldehyde 3-phosphate dehydrogenase.

Table S7. PCR primers used in the study.

Primer	Forward (5'→3')	Reverse (5'→3')
Dcytb	TCCTGAGAGCGATTGTGTTG	TTAATGGGGCATAGCCAGAG
DMT1	GCTGAGCGAAGATACCAGCG	TGTGCAACGGCACATACTTG
hephaestin	GGCACAGTTACAGGGCAGATG	ACATGGTCAGTAACGTGGCAGT
FPN	TTCCGCACTTTTCGAGATGG	TACAGTCGAAGCCCAGGAC
ferritin light chain	CACTCTTCCAGGATGTGCAG	ACAGAGGTGAGGGTCTGTGC
hepcidin	AGACACCAACTTCCCCATATG	ACAGAGACCACAGGAGGAATTCT
GAPDH	GTATCGGACGCCTGGTTAC	CTTGCCGTGGGTAGAGTCAT

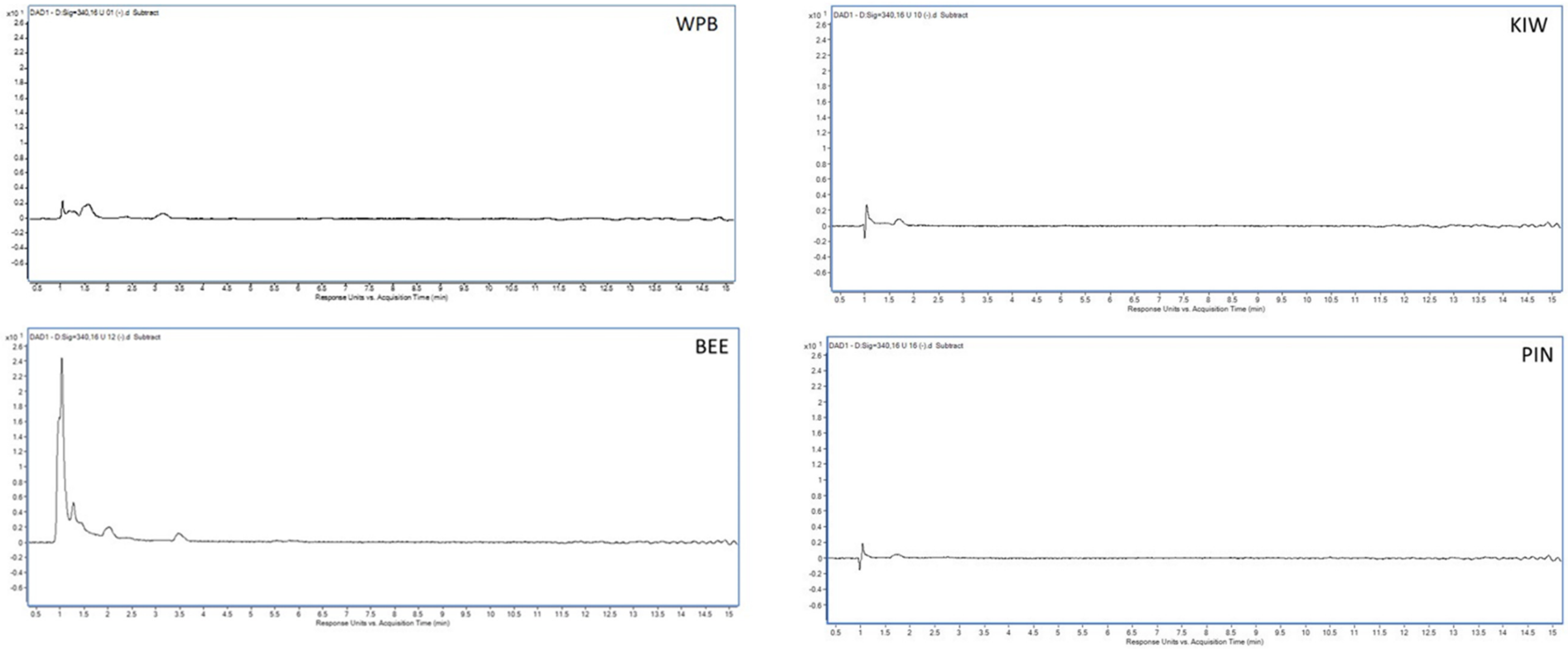
DAD1 - D:Sig=340,16 U 01 (-).d Subtract
WPB
Response Units vs. Acquisition Time (min)
DAD1 - D:Sig=340,16 U 10 (-).d Subtract
KIW
Response Units vs. Acquisition Time (min)
DAD1 - D:Sig=340,16 U 12 (-).d Subtract
BEE
Response Units vs. Acquisition Time (min)
DAD1 - D:Sig=340,16 U 16 (-).d Subtract
PIN
Response Units vs. Acquisition Time (min)

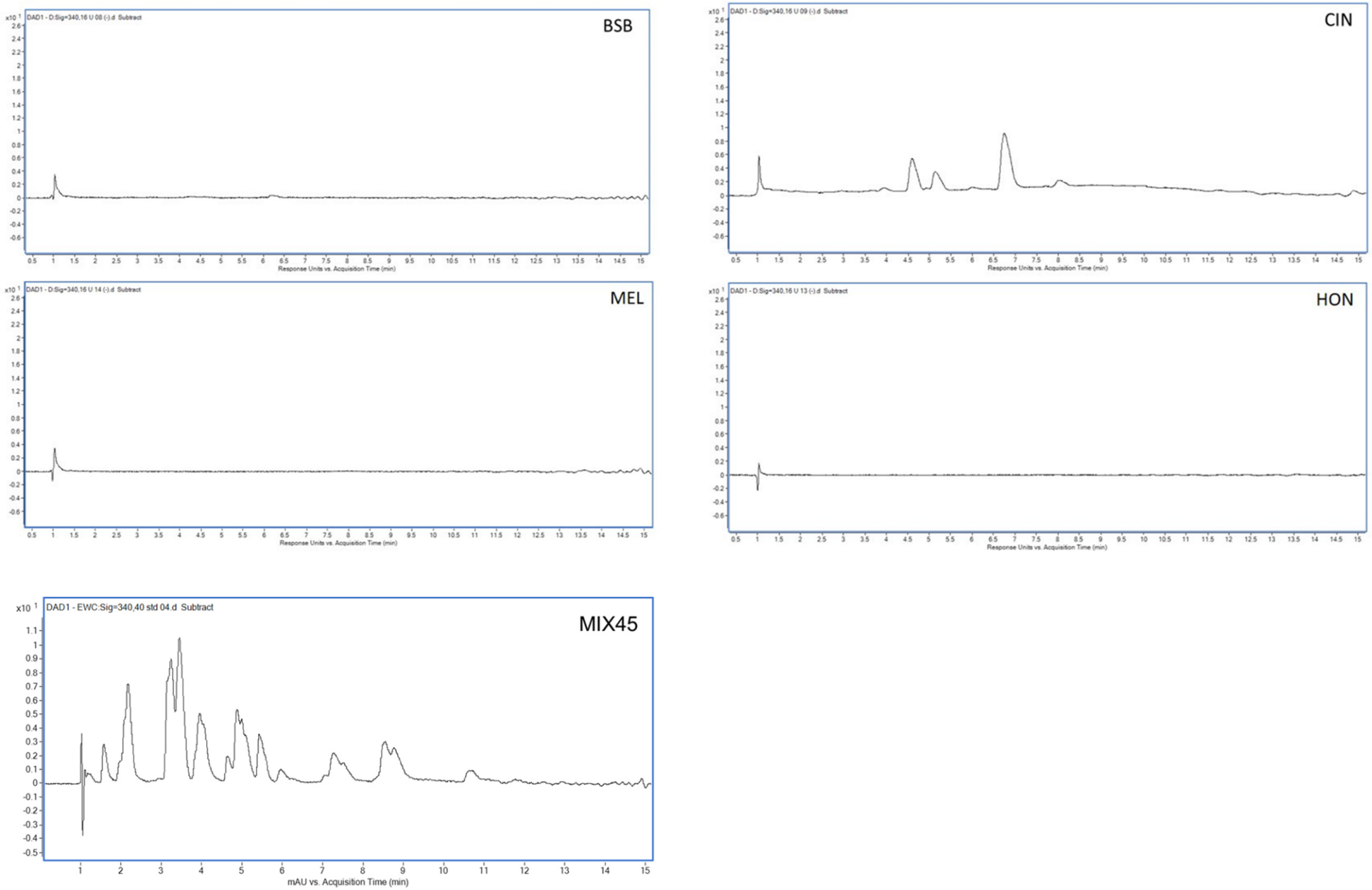

Figure S1. The HPLC-DAD chromatograms of polyphenolic compounds from selected plant-based food stuffs at 340 nm

BEE: beetroot cold-pressed juice; BSB: butternut squash boiled; CIN: cinnamon powder; HON: acacia honey; KIW: kiwi cold-pressed juice; MEL: melon cold-pressed juice; MIX45: mix of 45 compounds used as standards for quantitative analysis, concentration 3125 ng/mL; PIN: pineapple cold-pressed juice; WPB: white potato boiled.

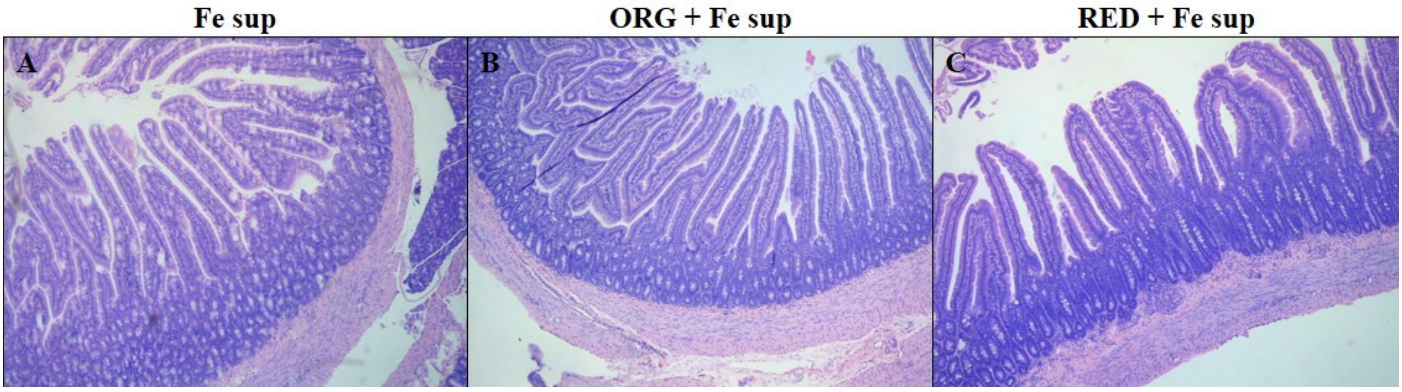

Figure S2. Photomicrographs of duodenal tissue of rats with IDA after simultaneous treatment with nutraceuticals (ORG and RED) and iron supplementation (H&E, 5×). A - Fe sup; B - ORG + Fe sup; C - RED + Fe sup. The duodenal structure in all three groups exhibited well-preserved layered wall composition and the distinctive villous morphology of the mucosa. The epithelium of the intestinal villi was maintained, displaying characteristics typical of intestinal tissue. Variability in mucosal appearance was observed in the height of the villi, with slightly reduced villi height noted in the Fe sup and ORG + Fe sup groups.

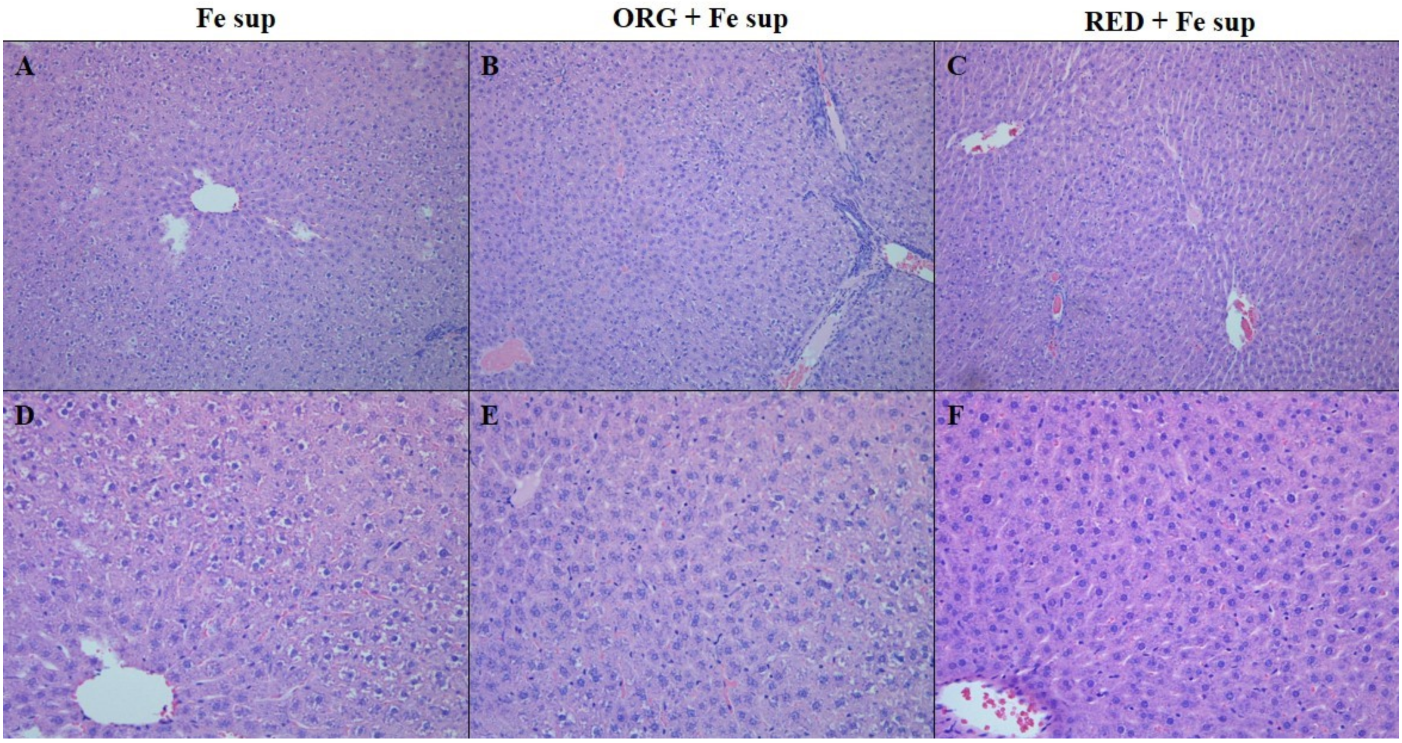

Figure S3. Photomicrographs of liver tissue of rats with IDA after simultaneous treatment with nutraceuticals (ORG and RED) and iron supplementation (H&E, 10× (A-C), 20× (D-F)). A, D – Fe sup; B, E – ORG + Fe sup; C, F – RED + Fe sup. The hepatic tissue in Fe sup group had a typical lobular structure (Figure S3A and S3D) with plates of hepatocytes radiating from the central vein to the periphery of the lobule. The hepatocyte cell borders were preserved, but cytoplasm exhibited signs of perinuclear halo and hydropic degeneration in 45.5 % of animals. Nuclei had regular shape

and size. Within the portal spaces, scant connective tissue was present, containing blood vessels and bile ducts, along with some lymphocytes. Small clusters of lymphocytes were found in lobules, among hepatocytes. The overall structure of the liver in ORG + Fe sup group maintained regular structure, with recognizable lobules featuring a characteristic shape (Figure S3B and S3E). Hepatic plates radiated from the central veins, while sinusoids were discernible and unimpaired. Hepatocytes exhibited a perinuclear halo and signs of hydropic degeneration, indicating damage of the cellular ultrastructure (Figure S3E). In comparison to Fe sup group, hepatocytes displayed slightly more perinuclear halo and hydropic degeneration, placed predominantly at the periportal area. Cell boundaries were clearly defined, while nuclei showed no indications of changes associated with cell death. In 37.5 % of animals, small clusters of lymphocytes were found among hepatocytes, and only in the tissue of one animal (12.5 %) a small focus of necrosis was present. The portal spaces contained limited connective tissue housing elements of the portal triad. The liver tissue of the animals in RED + Fe sup group preserved normal lobular arrangement (Figure S3C and S3F). Hepatic plates radiated from central veins, containing hepatocytes which, in comparison to Fe sup and ORG + Fe sup groups, exhibited similar occurrence of perinuclear halo (71.43 % of animals) but the signs of hydropic degeneration was absent. Nuclear changes indicating cell death (karyopyknosis, karyorrhexis) were absent. Lymphocytic inflammatory infiltrate was rarely seen in the lobules (28 % of animals), and necrosis was absent. The portal spaces contained scarce connective tissue where elements of the portal triad were embedded.

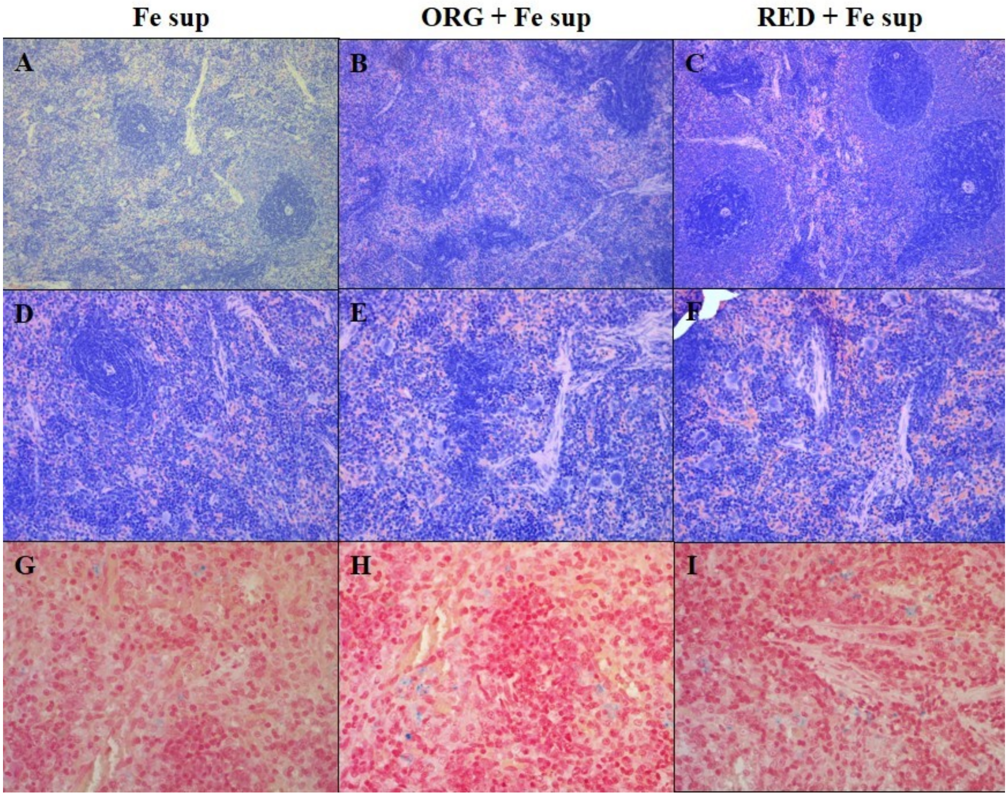

Figure S4. Photomicrographs of spleen section of rats with IDA after simultaneous treatment with nutraceuticals (ORG and RED) and iron supplementation (H&E, 10× (A-C), 20× (D-F); Prussian blue, 40× (G-I)). A, D, G – Fe sup; B, E, H – ORG + Fe sup; C, F, I – RED + Fe sup. In the histological analysis of the spleen of the Fe sup group, both red and white pulp were distinguishable (Figure S4A). The white pulp was preserved in 50 % of animals, while in others it was significantly reduced to a narrow band of lymphoid tissue around the centrofollicular artery (Figure S4A and S4D). The surrounding mantle zone was also notably reduced and in some cases absent. Opposite to white, the red pulp was more extensive and voluminous. Red pulp contained megakaryocytes, indicating the extramedullary hematopoiesis (Figure S4D). In 16.7 % of animals, megakaryocytes were abundant,

while in 83.3 % they were detectable but rare. The Prussian blue method (Figure S4G), revealed iron deposits evident as greenish-bluish cytoplasmic staining in a limited number of cells (7 cells per 10 high power fields (HPF)). This limited number of iron-containing cells, suggested iron depletion in the spleen of this group. Spleen of ORG + Fe sup group of animals (Figure S4B) had a regular appearance in 75 % of animals, and only a quarter of cases had reduced, impaired appearance of white pulp. The red pulp was notably pronounced and voluminous. In 75 % of cases megakaryocytes were numerous, indicative of the intense extramedullary hematopoiesis (Figure S4E). Utilizing the Prussian blue staining method, an increased number of cells with greenish-bluish dotted staining (11/10 HPF) was observed compared to Fe sup group, suggesting an increase in iron deposition due to the applied treatment (Figure S4H). Figure S4C exhibited the histological appearance of spleen of RED + Fe sup group. The white pulp was reduced in only 29 % of animals, less than in Fe sup and ORG + Fe sup groups. The red pulp was moderately voluminous, with frequent megakaryocytes in 86 % of animals, which is much more compared to other groups (Figure S4F). Following the application of the Prussian blue staining method (Figure S4I), an increase in iron-containing cells (16/10 HPF) was evident, and this increase was more pronounced compared to Fe sup and ORG + Fe sup groups. It can be inferred that the applied treatment led to an increase in the iron depot.

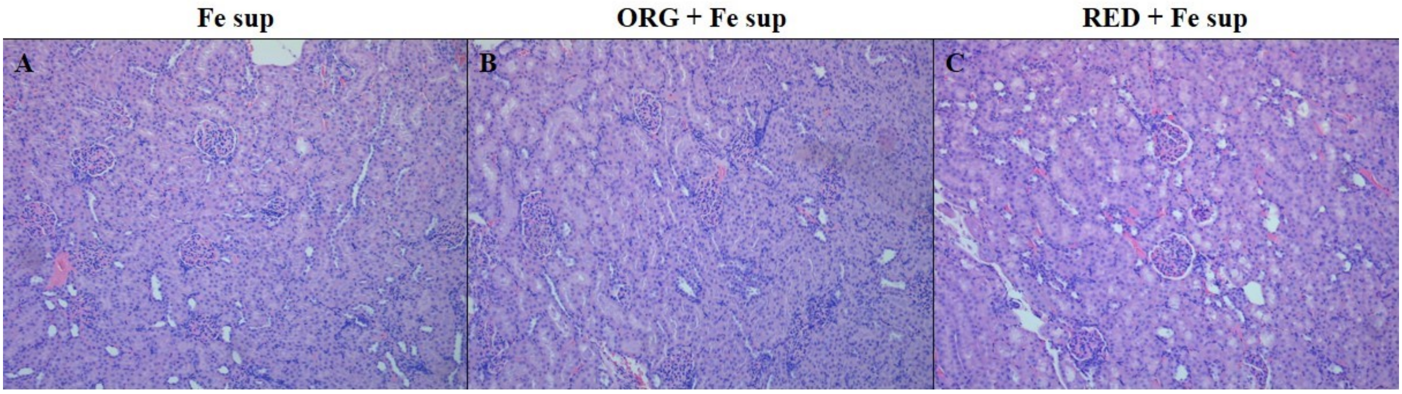

Figure S5 Photomicrographs of kidney section of rats with IDA after simultaneous treatment with nutraceuticals (ORG and RED) and iron supplementation. (H&E, 10×). A - Fe sup; B - ORG + Fe sup; C - RED + Fe sup. In Fe sup group samples of kidney tissue, both the cortex and medulla were identifiable, with the most of tissue damage was detected in cortex. Renal corpuscles are not reduced in number, but 25 % of them (semiquantitative assessment) had impaired structure (reduced size, narrow or absent Bowman's space) (Figure S5A). The tubular epithelium did not exhibit vacuolization in the tubular epithelial cells. In the kidney tissues of ORG + Fe sup group, the cortex and medulla were clearly distinguishable, with the most notable changes occurring in the kidney cortex. The number of renal corpuscles in the cortex does not appear to be reduced. In contrast to Fe sup group, a slightly smaller subset of renal corpuscles displayed a reduced size and narrow or absent Bowman's space. There was no observable vacuolization in the tubular epithelial cells (Figure S5B). Tissue samples of the kidney obtained from RED + Fe sup group exhibited the least tissue impairment. Cortex and medulla were clearly demarcated, with preserved number of renal corpuscles. Only about 10 % showed impairment in structure, while tubular structure and epithelium was intact (Figure S5C).

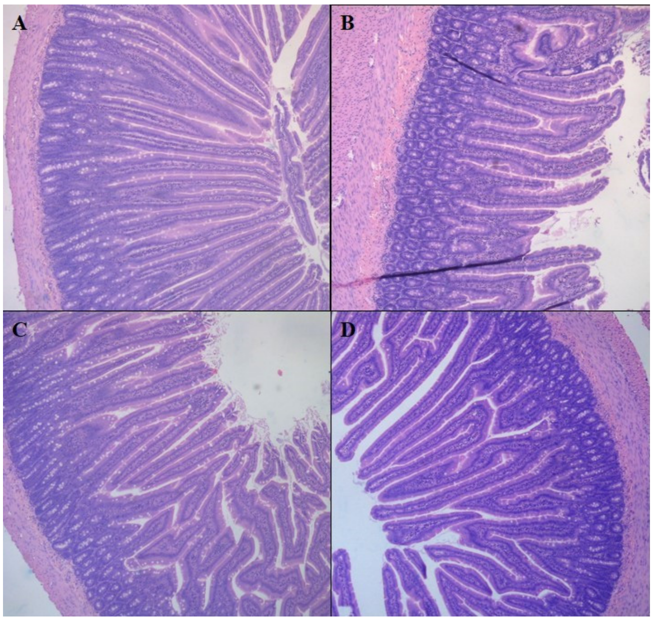

Figure S6. Photomicrographs of duodenal tissue of rats with IDA after treatment with nutraceutical ORG alone and enriched with iron (H&E, 5×). A - Normal, B - Control, C - ORG, D - ORG + Fe enrich. In the structural examination of the duodenum, the layered structure of the wall remained intact in all experimental groups. The thickness of the mucosa, in ORG and ORG Fe +enrich groups (C and D) was comparable to that of the Normal group (A), as opposed to Control group (B) where it was thinner. In all groups mucosa exhibited preserved villous structure. The intestinal villi, lined by intact intestinal epithelium, exhibited proper morphology, alignment and height. Intestinal glands (crypts of Liberkin) were present in adequate numbers and exhibited a regular arrangement, while the lamina propria contained a typical mononuclear inflammatory infiltrate.

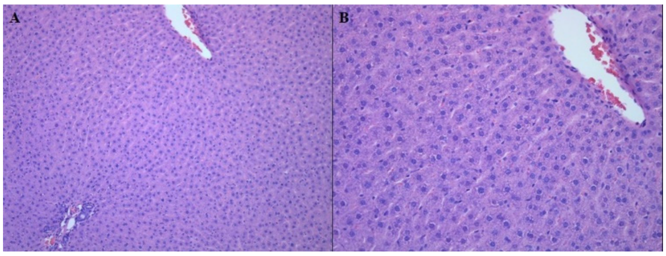

Figure S7. Photomicrographs of liver tissue of rats from Normal group: A - Preserved structure of the lobule, devoid of inflammatory infiltrate both within the lobule and the portal space (H&E, 10×); B - hepatocyte structure exhibited maintained integrity (H&E, 20×). The hepatic tissue in Normal group exhibited a typical structure, characterized by lobules with distinctive hexagonal shapes. One cell thick plates of hepatocytes radiate from the central vein to the periphery of the lobule, as do sinusoids. The hepatocytes displayed well-defined cell borders, homogeneous and abundant cytoplasm, and one to two nuclei, each featuring prominent nucleoli. Within the portal spaces, there is scant connective tissue containing blood vessels and bile ducts along with rare lymphocytes.

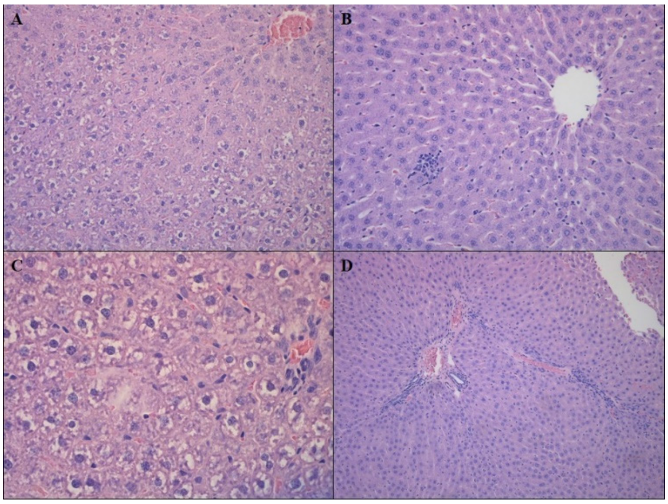

Figure S8. Photomicrographs of liver tissue of rats from Control group: A - hepatocytes exhibited periportal damage (H&E, 10×), B - inflammatory infiltrate observed in the lobules (H&E, 20×), C - evident pyknosis of hepatocyte nuclei (H&E, 10×). D - inflammatory infiltrate present in portal spaces (H&E, 20×). The overall architecture of the liver in Control group of anaemic rats was maintained, with recognizable lobules featuring a characteristic shape. Hepatic plates radiated from the central veins, centrally positioned within each lobule. Plates were separated by sinusoids which were well visible, displaying a slightly narrower lumen. Hepatocytes exhibited a pronounced perinuclear halo and signs of hydropic degeneration, indicating damage of the cellular ultrastructure (A). In some areas of the lobule, cell boundaries were indistinct, and individual nuclei displayed signs of karyopyknosis (C), suggesting a process of cell death. Focal regions within the lobules revealed clusters of inflammatory cells, including lymphocytes and neutrophilic granulocytes (B). In the portal spaces at the lobule corners, there was sparse to moderately abundant connective tissue containing elements of the portal triad (interlobular artery, interlobular vein and interlobular bile duct). A moderately abundant mononuclear inflammatory infiltrate, composed of lymphocytes and granulocytes, was evident in the connective tissue of the portal spaces (D). Notably, a significant number of portal space blood vessels appeared dilated and filled with blood.

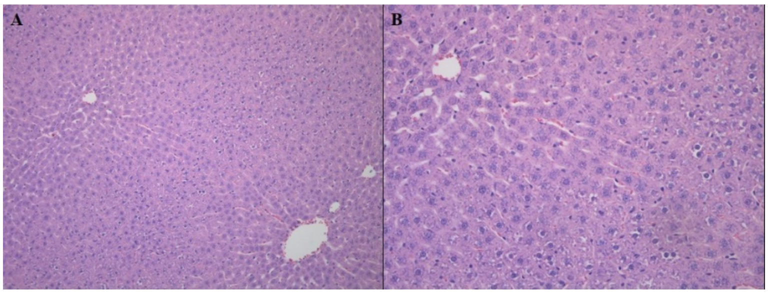

Figure S9. Photomicrographs of liver tissue of rats from ORG group: A - the preserved structure of liver lobules without inflammatory infiltrate in both the lobule and portal space (H&E, 10×); B - mild perinuclear halo observed in hepatocytes within the periportal zone of the lobulus, without nuclear pyknosis (H&E, 20×). The overall structure of the liver in ORG group remained intact. In comparison to Control group, hepatocytes displayed a notably reduced occurrence of perinuclear halo and hydropic degeneration, primarily evident at the lobule's periphery (periportal), while mild hepatocyte edema was observed in the remainder of the lobule. Cell boundaries were clearly defined, and nuclei exhibited no signs of karyopyknosis or karyorrhexis, indicating the absence of processes associated with cell death. Rare blood vessels within the portal spaces appeared dilated and filled with blood. The portal spaces contained limited connective tissue housing elements of the portal triad, and a mononuclear inflammatory infiltrate was within physiological limits.

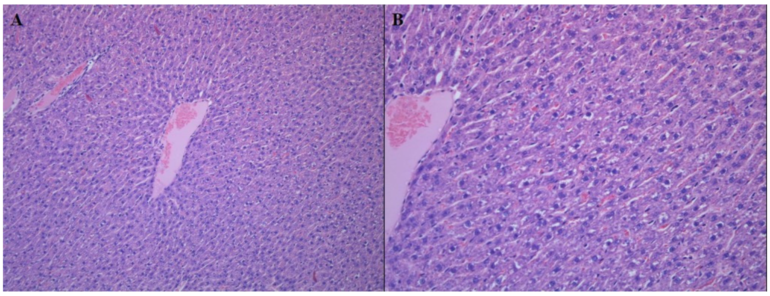

Figure S10. Photomicrographs of liver tissue of rats from ORG + Fe enriched group: A – preserved structure of liver lobules without any inflammatory infiltrate in both the lobule and portal space (H&E, 10×); B - individual hepatocytes exhibited a subtle perinuclear halo, and no nuclear pyknosis was evident (H&E, 20×). The liver tissue of the animals in ORG + Fe enrich group remained intact. In comparison to Control and ORG groups, hepatocytes exhibited a significantly reduced occurrence of perinuclear halo, while hydropic degeneration was absent, along with the absence of signs indicating cell death (karyopyknosis, karyorrhexis). The liver structure closely resembled that of the Normal group, with no dilation observed in the portal space vessels. The portal spaces contained scarce connective tissue housing elements of the portal triad and a mononuclear inflammatory infiltrate within physiological limits. It can be deduced that the administered treatment has effectively mitigated liver damage caused by IDA, as evidenced by the close resemblance of the liver tissue to that of the Normal group.

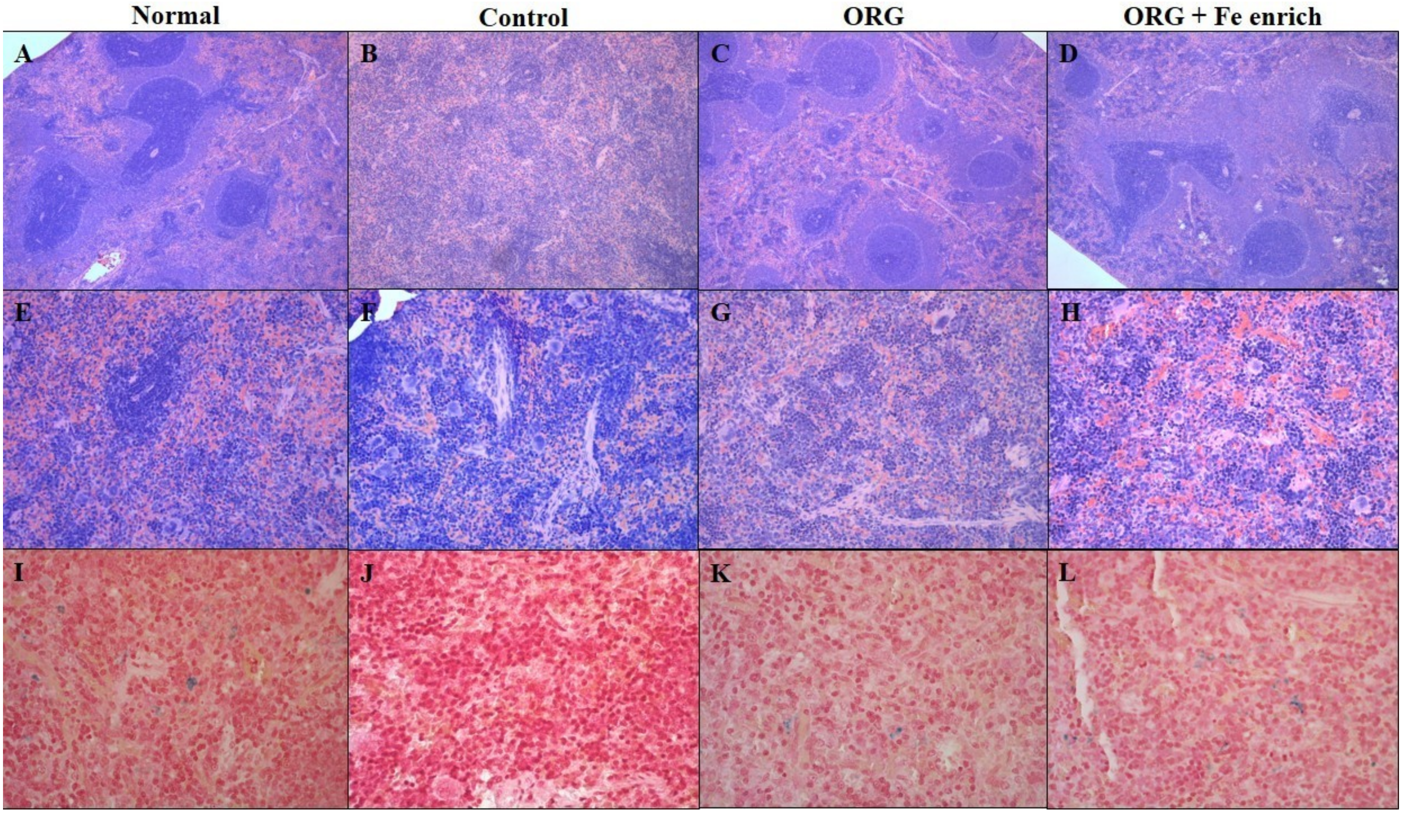

Figure S11. Photomicrographs of spleen tissue of rats with IDA after treatment with nutraceutical ORG alone and enriched with iron A-D: Alterations of white and red pulp (H&E, 5×); E-H: e presence of megakaryocytes, serving as an indicator of extra medullary haematopoiesis (H&E, 20×); I-L: Visualization of iron depots within spleen cells detected as a blue-green hue in cytoplasm, Prussian blue staining (40×). In the histological image of the spleen of Normal group, both red and white pulp were distinguishable. The white pulp was prominently developed around the centrofollicular arteries, featuring a broad mantle zone (A). The red pulp was moderately expressed, and upon H&E

staining, no evidence of extramedullary hematopoiesis was observed, with the absence of megakaryocytes (E). Utilizing the Prussian blue staining method (I), sporadic individual cells are identified, characterized by intensely blue-stained cytoplasm indicating of substantial iron depots. In the histological image of the spleen from Control group, both red and white pulp were identifiable. In comparison to the Normal group, the white pulp was diminished to a narrow band of lymphoid tissue around the centrofollicular artery (B). The surrounding mantle zone was also notably reduced. Conversely, the red pulp was accentuated and voluminous. Figure F revealed the presence of numerous megakaryocytes in the red pulp, indicative of the extramedullary hematopoiesis. With the application of the Prussian blue method (J), iron deposits were evident as greenish-bluish cytoplasmic staining in a limited number of cells (2/10 HPF). This limited number of iron-containing cells, suggested pronounced iron depletion in the spleen of this group. In the spleen of ORG group animals (C) the white pulp appeared reduced compared to Normal group, however, when compared to Control group, the white pulp and mantle zone were more clearly defined and more abundant. The red pulp was notably pronounced and voluminous, featuring numerous megakaryocytes indicative of the extramedullary hematopoiesis (G). Utilizing the Prussian blue staining method, an increased number of cells with greenish-bluish dotted staining (7/10 HPF) was observed compared to Control group, suggesting a rise in the iron pools due to the applied treatment (K). Similarly, in Figure D, the histological image reveals recognizable red and white pulp of ORG + Fe enrich group. The white pulp exhibited no discernible differences compared to the Normal group. When compared to Control group, both the white pulp and mantle zone were more distinctly defined and abundant. The red pulp was moderately expressed, with noticeable signs of extramedullary hematopoiesis (H). Employing the Prussian blue staining method (L), an increase in iron-containing cells (20/10 HPF) was evident, and this increase was more pronounced compared to Control group as well as ORG group. It can be inferred that the applied treatment led to an increase in the iron depot.

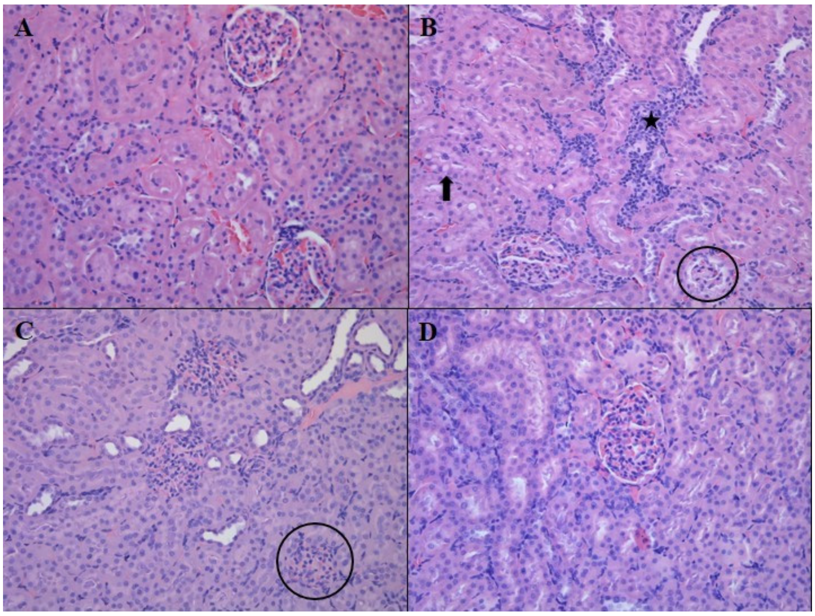

Figure S12. Photomicrographs of kidney tissue of rats with IDA after treatment with nutraceutical ORG alone and enriched with iron (H&E, 20×). A – Normal group displaying a preserved histological appearance of the kidney cortex; B – Control group exhibiting a reduced number of glomerular bodies, significantly smaller in size (circled), accompanied by marked peritubular infiltrate (asterisk) and vacuolization of the tubular epithelium (arrow); C - ORG group showcasing glomerular corpuscles slightly smaller in size (rounded) with no detectable Bowman's space, and an absence of peritubular infiltrate and vacuolization of the tubular epithelium; D - ORG + Fe enrich group depicting a histological appearance of the cortex without discernible differences when compared to the Normal group.

REFEREN⚜ES

58. Taconic Biosciences, https://www.taconic.com/rat-model/wistar-hannover-galas, (Accessed on November 15, 2023.)
59. Taconic Biosciences, *Control Data of BrlHan:WIST@Tac (GALAS) Rat*, Albany NY, 2003.
60. Charles River Laboratories, *Clinical laboratory Parameters for Crl:WI (Han)*, Wilmington MA, 2008.
61. Moshtaghie, M., Malekpouri, P., Dinko, M. R., Moshtaghie, A. A. Changes in serum parameters associated with iron metabolism in male rat exposed to lead, *J Physiol Biochem*, **2013**, *69*, 297–304. https://doi.org/10.1007/s13105-012-0212-9
62. He, H., Huang, Q., Liu, C., Jia, S., Wang, Y., An, F., Song, H. Effectiveness of AOS-iron on iron deficiency anemia in rats, *RSC Adv*ances, **2019**, *9*, 5053–5063. https://doi.org/10.1039/C8RA08451C
63. Moreno-Fernandez, J., Díaz-Castro, J., Alférez, M. J. M., López-Aliaga, I. Iron deficiency and neuroendocrine regulators of basal metabolism, body composition and energy expenditure in rats. *Nutrients*, **2019**, *11*, 631. https://doi.org/10.3390/nu11030631
64. Gravesen, E., Hofman-Bang, J., Mace, M. L., Lewin, E., Olgaard, K. High dose intravenous iron, mineral homeostasis and intact FGF23 in normal and uremic rats. *BMC Nephrology*, **2013**, *14*, 281. https://doi.org/10.1186/1471-2369-14-281
